# Microtubule dynamics is a therapeutic vulnerability in VHL-deficient renal cell carcinoma

**DOI:** 10.7150/ijbs.109642

**Published:** 2025-04-28

**Authors:** Yue Pu, Ziruoyu Wang, Shishi Tao, Eun Ju Yang, Songlin Wu, Guowen Ren, Li-Jie Chen, Xiumei Zhang, Kaeling Tan, Yongjun Dang, Joong Sup Shim

**Affiliations:** 1 Cancer Centre, Faculty of Health Sciences, University of Macau, Taipa, Macau SAR, China.; 2 Basic Medicine Research and Innovation Center for Novel Target and Therapeutic Intervention, Ministry of Education, College of Pharmacy, Chongqing Medical University, Chongqing, China.; 3 Institute of Cancer Research, Shenzhen Bay Laboratory, Shenzhen 518132, China.; 4 Basic Medicine Research and Innovation Center for Novel Target and Therapeutic Intervention, Ministry of Education, College of Pharmacy, The Second Affiliated Hospital of Chongqing Medical University, Chongqing Medical University, Chongqing, China.; 5 Ministry of Education Frontiers Science Centre for Precision Oncology, University of Macau, Taipa, Macau SAR, China.

**Keywords:** renal cell carcinoma, VHL, synthetic lethality, microtubule, drug target

## Abstract

Von Hippel-Lindau (VHL) is a tumor suppressor frequently mutated in renal cell carcinoma (RCC) and its loss has been considered as a target for therapeutic exploitation. In an effort to identify therapeutic vulnerabilities in VHL-deficient RCC, we found that SKPin C1, a SKP2 inhibitor, exhibited synthetic lethal effects on VHL-deficient RCC cells. SKPin C1 selectively disrupted spindle assembly in VHL-deficient RCC, leading to the induction of mitotic arrest and death. These effects were independent of its inhibitory action on SKP2. Our in-depth biochemical and molecular interaction studies reveal that SKPin C1 binds to tubulin and inhibits microtubule polymerization. Interestingly, anti-microtubule effect of SKPin C1 was much more pronounced in VHL-deficient RCC cells. Further mechanistic studies on the synthetic lethality reveal that VHL loss alters microtubule dynamics in cells, promoting microtubule growth speed while reducing stability. Treatment of VHL-deficient RCC cells with SKPin C1 or other microtubule destabilizers strongly suppressed microtubule growth and reduced the levels of GTP-tubulin and acetylated microtubules, resulting in selective vulnerability in VHL-deficient RCC. Taken together, our study suggests that microtubule dynamics is a therapeutic vulnerability in VHL-deficient RCC and provides a rationale for the combination treatment of VHL-deficient RCC with anti-microtubule agents and RCC targeted therapies.

## 1. Introduction

Kidney cancer is amongst the ten most prevalent cancers worldwide, and renal cell carcinoma (RCC) is the most common type, accounting for more than 90% of kidney cancer^1^. RCC is hard to treat because it is largely insensitive to radiotherapy and chemotherapy^2^-^4^, and the surgery is only suitable for patients with early stage, localized tumors. Conventional immunotherapy cytokines, such as interferon and high-dose interleukin-2 have been used as first-line treatments for patients with metastatic RCC, but they usually benefit only a small fraction of patients and have significant toxicity^5^. Recent developments in targeted therapy and immunotherapy, such as receptor tyrosine kinase inhibitors and immune checkpoint blockades, have largely benefitted patients with late stage RCC. However, several challenges have appeared with these treatment options, including tumor acquisition of drug resistance against targeted therapy drugs^6^. For immune checkpoint blockades, it is difficult to predict the RCC patients' response to the drugs due to lack of reliable predictive biomarkers^7^. Therefore, development of more personalized treatment approach with predictable biomarkers is needed for patients with RCC.

Loss of von Hippel-Lindau tumor suppressor (VHL) as a result of gene mutation or epigenetic silencing appears in more than half of entire RCC patients, making it a crucial biomarker for RCC^8^. VHL has been well characterized with its functions as a component of the E3 ubiquitin ligase complex in oxygen-dependent ubiquitination and degradation of hypoxia-inducible factor α (HIF-α)^9^-^11^. Under normal oxygen conditions, HIF-α is hydroxylated by prolyl hydroxylase (PHD), recognized by the multimeric E3 ubiquitin ligase complex containing VHL, and targeted for ubiquitination and proteasomal degradation. Loss of VHL leads to the stabilization and accumulation of HIF-α even under normoxia, promoting the dimerization with a HIF-β subunit and the translocation into the nucleus where it binds to the hypoxia-response elements (HREs) to initiate the transcription of target genes, such as VEGF, GLUT1, EPO and PDGF. The elevation of the HIF target genes in VHL-deficient RCC leads to enhanced tumor angiogenesis and glucose metabolism, characteristic features of RCC^12^, ^13^. In addition to its function in controlling HIF-dependent transcription, VHL is known to have several HIF-independent, non-canonical functions in various biological pathways, such as extracellular matrix (ECM) assembly, microtubule stability and primary cilia maintenance and WNT signaling via interacting with multiple protein partners^14^, ^15^. While a number of these non-canonical functions of VHL in RCC initiation and development are subjects for further investigation, it has been well recognized that VHL loss is an important biomarker for targeted therapy development in RCC.

In order to discover biomarker-driven, therapeutic vulnerabilities in RCC, we employed a synthetic lethal drug screen in VHL-deficient RCC cells. Synthetic lethality is an approach to leverage the cancer cell dependency occurring when a tumor suppressor is inactivated^16^. This approach has been actively exploited in cancer target discovery and personalized therapy since the successful clinical introduction of PARP inhibitors for the treatment of BRCAness cancers^17^, ^18^. VHL loss has also been a hot topic in synthetic lethality approach, where several potential synthetic lethal targets or compounds have been discovered. The natural product Englerin A, which selectively inhibits the VHL-deficient cells by stimulating PKC θ and causing the metabolic disaster^19^. Besides, inhibition of EZH1^20^ or TBK1^21^ could specifically inhibit RCC cell growth by inducing synthetic lethality with VHL loss. To further expand VHL biomarker-driven drug targets for RCC, we conducted a synthetic lethal drug screen for VHL against highly-selective inhibitor library containing 318 small molecule inhibitors targeting various cellular druggable proteins. SKPin C1, a small molecule inhibitor of the E3 ubiquitin ligase SKP2, was amongst the top candidates of synthetic lethal drugs identified in VHL-deficient RCC cells. Mechanistic deconvolution of the synthetic lethal effect of SKPin C1 on VHL-deficient RCC cells, however, revealed that SKPin C1 inhibited microtubule dynamics in the cells in a SKP2-independent manner. VHL-deficient RCC cells were hypersensitive to microtubule inhibitors through the disruption of microtubule-spindle assembly and the induction of mitotic arrest and death. Our study not only demonstrated previously unrecognized target of SKPin C1 but also provided a novel therapeutic strategy for the treatment of RCC with VHL loss.

## 2. Materials and methods

### 2.1. Cell lines and culture

786-O was obtained from American Type Culture Collection (ATCC, Manassas, VA), 769-P and Caki-1 were obtained from the National Collection of Authenticated Cell Cultures (Shanghai, China). All the cell lines were authenticated by Short Tandem Repeat (STR) profiling (Applied Biosystems, Foster, CA). 786-O and 769-P are maintained in Gibco RPMI 1640 (Thermo Fisher Scientific, Waltham, MA) with 10% fetal bovine serum (FBS, Thermo Fisher Scientific), 1% Penicillin/Streptomycin (Thermo Fisher Scientific) and 1% Sodium Pyruvate (Thermo Fisher Scientific). Caki-1 is maintained in MyCoy's 5A medium with 10% FBS and 1% Penicillin/Streptomycin. All cells were cultured under 37 °C with 5% CO2 in the humidified incubator.

### 2.2. Stable overexpression of plasmid in cells

786-O cells were seeded into the 12 well plates. When cell confluence is about 80%, separately prepared A solution: dilute 3μl of Lipofectamine 3000 (Thermo Fisher Scientific) in 35μl Opti-MEM (Thermo Fisher Scientific), then the B solution: 1 μg plasmid (HA-VHL-pRc/CMV plasmid, Addgene, #19999 or EB1 plasmid, Addgene, #39299) in 35 μL Opti-MEM, mixed them and incubated 15 min at room temperature, after that add the mixture to cell drop by drop. The untransfected cells were eliminated with 2 mg/ml neomycin (sc-29065B, Santa Cruz Biotechnology, Dallas, TX), and the rest of the live cells were seeded into the 96-well plates at a density of 1 cell per well, so that each well has only one cell to form the single colony. 12 days later, trypsinize and split the single clone for expansion and verification of VHL or EB1 expression.

### 2.3. Highly selective inhibitor library screening

Highly selective inhibitor library was purchased from Selleck Chemicals (Houston, TX), which consists of 318 compounds. Each compound was prepared in eight doses with three times dilution in 384-well plates. 786-O *VHL*^-/-^ cells and 786-O wt*VHL*^oe^ cells were seeded into two sets of libraries separately. After cultured in a 37°C incubator for 72 hours, perform the cell viability assay and calculate the half-maximal inhibitory concentration (IC50) using GraphPad Prism software 9.3.1 (GraphPad, Boston, MA). Selectivity Index (SI) was calculated for each drug toward wildtype and mutant cells based on following formula: SI = IC50 (786-O wt*VHL*^oe^)/ IC50 (786-O *VHL*^-/-^), and presented in the SI plot. For drugs that reach IC50 in both wildtype and mutant cell lines, accurate IC50 values were generated by GraphPad Prism software and their SI values were presented in the SI plot. For drugs that do not reach IC50 in both cell lines, we excluded these drugs from the SI analysis. For drugs that reach IC50 only in one cell line (either wildtype or mutant), we considered these drugs to be the best selective drugs in either synthetic lethality or synthetic viability. To present these drugs in the SI plot, we provided IC50 values in two ways: (1) If the inhibition curve on the non-effective cell line shows a declining trend albeit not reaching 50%, we used the theoretical IC50 value generated by GraphPad Prism software. (2) If the inhibition curve was flat and GraphPad Prism software could not calculate a theoretical IC50, we assigned an arbitrary IC50 value of 100 µM for the non-effective cell line (this value is five times greater than the maximum concentration (20 µM) used in the screen).

### 2.4. Cell viability assay

The cell viability was detected by Alamar Blue solution which contains 0.025% (w/v) resazurin sodium salt (Sigma-Aldrich, St. Louis, MO) dissolved in sterile PBS. Alamar Blue solution was directly added to the cell culture medium at a volume ratio of 1:10 and incubated under a 37°C incubator for 2-3 hours until there were significant color changes and differences. Fluorescence intensity was measured (Excitaion:560 nm / Emission: 590 nm) by microplate reader SpectraMax M5 (Molecular Devices, Sunnyvale, CA) to determine the cell viability.

### 2.5. Western blot

The whole cell proteins were extracted by the 2X Laemmli lysis buffer (62.5 mM Tris-HCl, pH 6.8, 10% glycerol, 1% SDS, 0.005% Bromophenol Blue, 10% 2-mercaptoethanol), then boiled at 95°C for 10 minutes. An equal amount of protein was loaded into each well of SDS-PAGE (polyacrylamide-electrophoresis) gels, and then transferred the gels which containing separated proteins to the polyvinylidene fluoride (PVDF) membrane. After blocking with 5% skim milk, the membranes were incubated with the specific primary antibodies at 4°C overnight, then washed 3 times with PBST and incubated with the corresponding HRP conjugated secondary antibody at room temperature for 2 hours. After washing 3 times with PBST, the membranes were exposed to ChemiDoc™ MP Imaging System (Bio-Rad, Hercules, CA) with enhanced chemiluminescent substrate (Thermo Fisher Scientific).

### 2.6. Reverse transcription and quantitative real-time PCR (RT-qPCR)

The total RNA was isolated from cells with RNeasy Plus Mini Kit (Qiagen, Germantown, MD), then reverse transcribed into cDNA with High-Capacity cDNA Reverse Transcription Kit (Thermo Fisher Scientific). After that, the reaction mixture was prepared by combining the components in a PCR tube, including the cDNA, the target gene primers, water, and Taq Universal SYBR Green Supermix (Bio-Rad), loaded into the CFX96 Real-Time PCR System (Bio-Rad) and underwent 40 amplification reactions. The cycle threshold (Ct) value for each sample was calculated to determine the relative mRNA expression level.

### 2.7. Small interfering RNA gene silencing

Reverse transfection of siRNA was used to knock down the specific genes and was performed three repeating wells in 96 well plates. For each well, the siRNA was diluted in 10 μL Opti-MEM and mixed with 10 μL Opti-MEM containing 0.1 μL Lipofectamine RNAi max reagent (Thermo Fisher Scientific), then incubated at room temperature for 15 min, 2000 cells per well were added into each well and incubate 48-72 hours, validate the knockdown efficiency of the target gene by Western blotting.

### 2.8. Cell cycle analysis

Cells were harvested and washed with PBS, fixed with 70% ethanol, and incubated at -20°C overnight. After washing with PBS, the cells were resuspended in the solution containing RNase A (20 μg/ml) and incubated for 30 minutes at 37°C. Subsequently, washing with PBS three times, cells were incubated in the propidium iodide (PI) (50 μg/ml) solution for 30 minutes at room temperature in the dark and then analyzed the cells using CytoFLEX Flow Cytometer (Beckman Coulter, Brea, CA).

### 2.9. Immunofluorescence

Cells were fixed with 4% paraformaldehyde (sc-281692, Santa Cruz Biotechnology) at 37°C in the incubator for 30 min, then permeabilized with 0.1% Triton X-100 in PBS. After being blocked with the 3% BSA solution at room temperature for 30 min, the cells were incubated with the specific primary antibody at 4°C overnight, then incubated with the corresponding secondary antibody conjugated with Alexa Fluor-488 or Alexa Fluor-598 (Thermo Fisher Scientific) at room temperature for 1 hour, and the cellular nuclei were stained with DAPI for 5 min. Then the cells were mounted with a mounting medium. The images were acquired with the Nikon A1R confocal microscope (Nikon Corporation, Japan). The intensity of fluorescence was analyzed with ImageJ.

For the immunofluorescence of GTP-tubulin which was modified according to the method in published article^22^. To minimize cell shedding during operation, cells were seeded in the chamber coated with 0.1mg/ml ploy-d-lysine. Cells were permeabilized in PEM buffer (80 mM PIPES, 2 mM EGTA, 1 mM MgCl2, pH 6.9) containing 10% glycerol and 0.1% Triton-X100 for 3 min at 37 ° C, then incubated with anti-Tubulin-GTP (MB11) (1:2000, AG-27B-0009, AdipoGen, San Diego, CA) diluted in PEM buffer containing 10% glycerol and 0.2% BSA at 37 ° C for 15 min. After twice washing with PEM-10% glycerol buffer, the cells were incubated with Goat anti-Human Alexa Fluor 488 (1:1000, A-11013, Thermo Fisher Scientific) diluted in PEM buffer containing 10% glycerol and 0.2% BSA at 37 ° C for 15 min. After that paraformaldehyde fixation followed as described above.

### 2.10. EB1 tracking

786-O VHL isogenic cells stably overexpressing EB1-GFP were seeded into the chamber separately and cultured with 300 ul normal growth media 24 hours in advance. For time-lapse image acquisition, the microscope incubator was exploited to maintain 37°C and 5% CO2. Imaging was performed every 1 second for 1 minute, exposure time is 200 ms per image, using a Nikon TiE Widefield Microscope equipped with a Nikon DS-QI2 camera (Nikon Corporation), using APO 60x/1.4 DIC Oil Objective, 488 excitation laser, and NIS software. The EB1 comets were analyzed with plusTip Tracker, which has been developed by Kathryn T. Applegate etc.^23^. For SKPin C1 treatment, 786-O VHL isogenic cells stably overexpressing EB1-GFP were seeded into the 8-well chamber (Thermo Fisher Scientific) separately and cultured with 300 μl normal growth media for 24 hours in advance. As microtubules would be destroyed and become fade after SKPin C1 treatment, we used Olympus SpinSR10 Spinning Disk Confocal Microscope (Olympus Corporation, Japan) to reduce the quenching of fluorescence and obtain the clear video. The microscope incubator was exploited to maintain 37°C and 5% CO2, then set the experiment program: 488 excitation laser, Olympus UPLXAPO 100X Oil Immersion Objective (Olympus Corporation), the imaging is acquired every 1 second for 1 minute with an exposure time of 200 ms, which is performed every 15 min for totally 8 times. Subsequently, 300 μl growth medium containing 10 μM SKPin C1 was added into the chamber, leading to a final concentration of 5 μM SKPin C1, and start the program immediately. Finally, the frames at 1, 15, 30, 45, 60, 75, 90, and 105 min after SKPin C1 addition were extracted and cropped from the video.

### 2.11. In-cell microtubule polymerization assay

To determine the amount of α-tubulin in soluble (S) and polymerized (P) fraction, the cells were suspended in extraction buffer containing 0.1 M Pipes, pH 7.1, 1 mM MgSO4, 1 mM EGTA, 2 M glycerol, 0.1% Triton X-100, and protease inhibitors cocktails (Sigma-Aldrich). After incubation for 15 min, the cell lysates were centrifuged at 15,000 rpm for 15 min. The supernatant (containing 0.1% Triton-soluble tubulin) was transferred into another 1.5-ml tube. The remaining pellet was resuspended in lysis buffer (25 mM Tris-HCl, pH 7.4, 0.4 M NaCl, and 0.5% SDS) and boiled for 10 min. The sample was centrifuged at 15,000 rpm for 5 min, and the polymeric tubulin-containing supernatant was collected. The 0.1% Triton-soluble and -insoluble solutions were subjected to SDS-PAGE and detected by Western blotting with anti-α-tubulin antibodies. GAPDH antibody was used as a loading control for each experimental condition. The microtubule polymerization status in each experimental condition was quantitated based on the relative amount of P out of total (P + S).

### 2.12. In vitro tubulin polymerization assay

The activity of the compounds in inhibiting microtubule polymerization in vitro was evaluated with the Tubulin polymerization assay kit (cat. #BK011P, Cytoskeleton, Denver, CO). The kit contains fluorescent reporter chemicals that are incorporated into polymerized tubulins during polymerization reaction. The fluorescence intensity increases as tubulin polymerizes, which can be quantified as a polymerization curve. According to the instructions, the assay components were mixed to make the reaction buffer which contains 2mg/ml tubulin, 80 mM PIPES pH 6.9, 2 mM MgCl2, 0.5 mM EGTA, 1 mM GTP, and 20% glycerol. 5 µl of DMSO or 10x compounds solution were pipetted into the 96 well plate (Cat. # 3686, Corning, Corning, NY) separately and warmed to 37°C for 1 minute. After that 50 µl reaction buffer was added to each well. The fluorescence intensity was recorded by SpectraMax M5 (Molecular Devices) at Ex. 360 nm and Em. 450 nm- every 30 sec for 90 min at 37 °C.

### 2.13. Biolayer Interferometry (BLI)

The binding affinities between compounds with tubulin protein were determined by a biolayer interferometry assay using Octet RED 2 (ForteBio, Fremont, CA). After prewetting for 10 minutes, the SSA biosensor tip (ForteBio) was immobilized with 20 µg/ml biotin-labeled tubulin proteins (T333P-B, Cytoskeleton) in GTP-General tubulin buffer (G-PEM) for 600 seconds. The G-PEM buffer was used as a working buffer to maintain the activity of tubulin protein which contains 80 mM PIPES pH 6.9, 2 mM MgCl2, 0.5 mM EGTA, and 1 mM GTP. At the same time, a duplicate biosensor tip was incubated in G-PEM buffer containing 0.2 µM biotin as background binding control. Then the two tips were inserted parallel into buffer wells or the wells contained the diluted drugs, executing the procedures: baseline 60s, association 90s, and dissociation 90s in turn. All assays were performed in 96-well black plates with a total volume of 250 μl/well at 22 °C. All the data were analyzed by Octet Data Analysis HT software 11.1 (ForteBio).

### 2.14. Tumor xenograft mouse model

All animal experiments were approved by the Animal Research Ethics Committee of the University of Macau. Six- to seven-week female athymic nude mice were used for tumor xenograft and maintained at the University of Macau, a specific pathogen-free (SPF) Animal Facility. 786-O *VHL*^-/-^ cells were suspended in Matrigel (Corning) and implanted in the right flanks of nude mice (5×10^6^ cells per mouse). When tumors were palpable, mice were randomized into 5 groups (n = 5 mice per group) and treated with vehicle alone (sterile saline containing 5% DMSO, 5% tween-80 and 5% polyethylene glycol-400), SKPin C1 (10 and 20 mg/kg), or Vinorelbine (2.5 and 5 mg/kg) by intraperitoneal (i.p.) injection once a day. Mice weight and tumor volume were measured every 5 days. Tumor volumes were calculated according to the following modified ellipsoid formula: long axis ×short axis^2^ × π/6. After 55 days, all the mice were euthanized and the tumor tissues were collected for weighing and stored in a liquid nitrogen tank for further analysis.

### 2.15. Statistical analysis

All experiments were repeated at least three times. Statistical significances were analyzed by Student's t-test or ANOVA analysis with GraphPad Prism software 9.3.1. The P value <0.05 was considered to be statistically significant.

## 3. Results

### 3.1. SKPin C1 induced synthetic lethality in VHL-deficient RCC cells in a SKP2-independent manner

**Figure 1:**
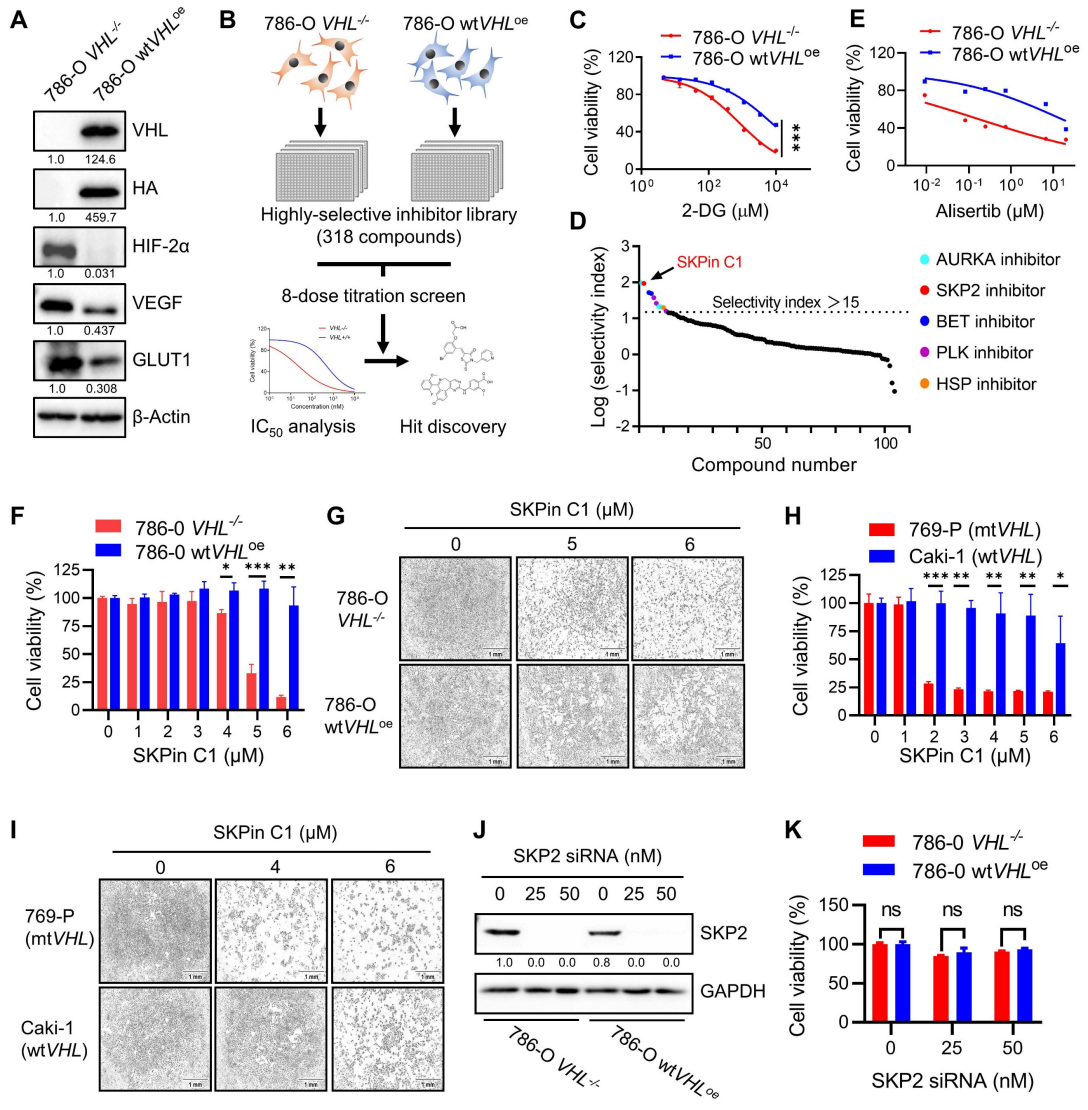
** SKPin C1 induced synthetic lethality in VHL-deficient RCC cells in a SKP2-independent manner. A** Verification of VHL-isogenic RCC cell line. Western blotting analysis of VHL, HA tag and its downstream target genes HIF-2α, VEGF, GLUT1 expression in VHL-isogenic RCC cell line. Data are shown as the mean ± SD, n=3. B Schematic illustration of highly selective inhibitor library screening for synthetic lethality. VHL-isogenic RCC cell pairs were parallelly seeded and treated with 318 selective inhibitors with 8-dose in 384-well plates, after 72 hours incubation, the cell viability was detected by alarm blue assay and analyzed the IC50 to identify the potential hits. **C** Dose response curve of 786-O VHL-isogenic cell pair treated with 2-DG- for 72 hours which has been already reported to induce synthetic lethality. Data are shown as the mean ± SD, n=3. ***P<0.001 between two indicated groups, two-way ANOVA. **D** Ranking of drugs according to the Log (Selectivity index). SI (Selectivity index) =IC50(786-O *VHL*^-/-^) / IC50(786-O wt*VHL*^oe^). SI>15 were identified as the synthetic lethal hits which were marked in colors. **E** Dose response curve of 786-O VHL-isogenic cell pair treated with Alisertib for 72 hours originated from screening result. **F-I** Validation of the SKPin C1 induced-synthetic lethality. VHL-isogenic RCC cell pair (F) and VHL non-isogenic RCC cell pair (H) were treated with SKPin C1 for 72 hours, the cell viability was detected by alarm blue assays. Data are shown as the mean ± SD, n=3. *P<0.05, **P<0.01, ***P<0.001 between two indicated groups, Student's t-test. (F). The representative images of cell density in VHL-isogenic RCC cell pair (G) and VHL non-isogenic RCC cell pair (I) treated with the indicated concentration of SKPin C1. **J-K** SKP2 inhibition on cell viability in VHL-isogenic cell pair. 786-O *VHL*^-/-^ and786-O wt*VHL*^oe^ cells were transfected with 25 nM, 50 nM SKP2 siRNA and incubated for 48 hours. Western blotting analysis of SKP2 protein level GAPDH was used as a loading control (J). The cell viability was evaluated by AlarmBlue assays. Data are shown as the mean ± SD, n=3. ns denotes not significant (K).

To screen and identify synthetic lethal targets of VHL in RCC, we first generated VHL-isogenic RCC cell pair using 786-O RCC cell line that has VHL loss-of-function mutation (786-O *VHL^-/-^* cells). We stably transfected HA-VHL plasmid into 786-O cells and established a VHL-stable overexpressing cell line (786-O wt*VHL*^oe^ cells) (Supplementary Fig. S1A-B). To verify the functional expression of wildtype VHL in 786-O wt*VHL*^oe^ cells, we analyzed the protein level of HIF-2α, a target protein of VHL E3 ubiquitin ligase complex, and HIF-2α transcriptional target genes, including VEGF and GLUT-1. The protein levels as well as mRNA levels of VEGF and GLUT-1 were all significantly decreased in 786-O wt*VHL*^oe^ cells compared to its isogenic parental 786-O *VHL^-/-^* cells (Fig. 1A; Supplementary Fig. S1C-E), verifying the functional VHL expression. We used the VHL-isogenic cell pair to screen synthetic lethal targets against a compound library, containing 318 highly selective inhibitors targeting various human druggable proteins. The screen was carried out in an 8-dose titration format to determine IC50 values of individual drugs against *VHL^-/-^* and wt*VHL*^oe^ cells (Fig. 1B). Synthetic lethal compounds that selectively inhibit VHL-deficient cells were identified based on the Selective Index (SI) value calculated by the following formula: SI= IC50 for 786-O wt*VHL*^oe^ / IC50 for 786-O *VHL^-/-^*. To examine the feasibility of our screening system, we tested the inhibitor of glucose metabolism 2-Deoxy-D-glucose (2-DG), a known drug inducing synthetic lethality in VHL-deficient cells^19^, in the 8-dose titration format. 786-O *VHL^-/-^* cells were significantly more sensitive to 2-DG than 786-O wt*VHL*^oe^ cells with an SI value of 9 (Fig. 1C). With this screening system, we identified 11 compounds as top candidate synthetic lethal compounds with SI values bigger than 15, including 4 AURKA (aurora kinase A) inhibitors, 1 SKP2 inhibitor, 2 BET (bromodomain and extra-terminal motif) inhibitors, 3 PLK1 (polo-like kinase 1) inhibitors, and 1 HSP (heat shock protein) inhibitor (Fig. 1D). Among the candidates, AURKA inhibitors including alisertib (Fig. 1E) appeared as most abundant hits. In fact, AURKA inhibitors have been reported to be more selective toward VHL-deficient cells^24^, reaffirming the feasibility of our screening system.

SKPin C1, a small molecule inhibitor of the E3-ubiquitin ligase SKP2, appeared as the second top candidate from the screen and was selected for further investigation for synthetic lethality with VHL in this study (Fig. 1D). SKPin C1 was originally developed as an inhibitor of the interaction between SKP2 and p27^KIP1^ by binding to the interaction interface^25^. The compound then inhibits SKP2-mediated degradation of p27 (and other CDK inhibitors such as p21^CIP1^) and causes cellular accumulation p27, inducing cell cycle arrest and apoptosis^26^. To verify the screening results, we tested the effect of SKPin C1 on the cell viability in VHL-isogenic 786-O cell pair and in VHL non-isogenic RCC cell pair, including 769-P (VHL mutant) and Caki-1 (VHL wildtype) cells. The result showed that SKPin C1 selectively inhibited the cell viability of VHL-deficient RCC cells compared to the VHL-wild type ones (Fig. 1F-I). Since SKPin C1 is an inhibitor of SKP2, we wondered whether SKP2 inhibition was responsible for the synthetic lethality with VHL. Silencing of SKP2 expression by specific siRNA could not recapitulate the synthetic lethal effect shown by SKPin C1 in VHL-deficient RCC cells (Fig. 1J-K). To verify the inhibitory effect of SKPin C1 on SKP2 in RCC cells we used, we analyzed the levels of p27 and p21 in 786-O VHL-isogenic cell pair treated with SKPin C1. SKPin C1 successfully increased the levels of p27 and p21 in both *VHL*^-/-^ and wt*VHL*^oe^ cells, verifying that SKP2 function was inhibited by the compound in both cells (Fig. S2). These results indicated that the synthetic lethal effect of SKPin C1 in VHL-deficient RCC cells was independent of its inhibitory effect on SKP2.

### 3.2. SKPin C1 increased mitotic cell population and up-regulated mitotic kinases and cyclins in VHL-deficient RCC cells

**Figure 2:**
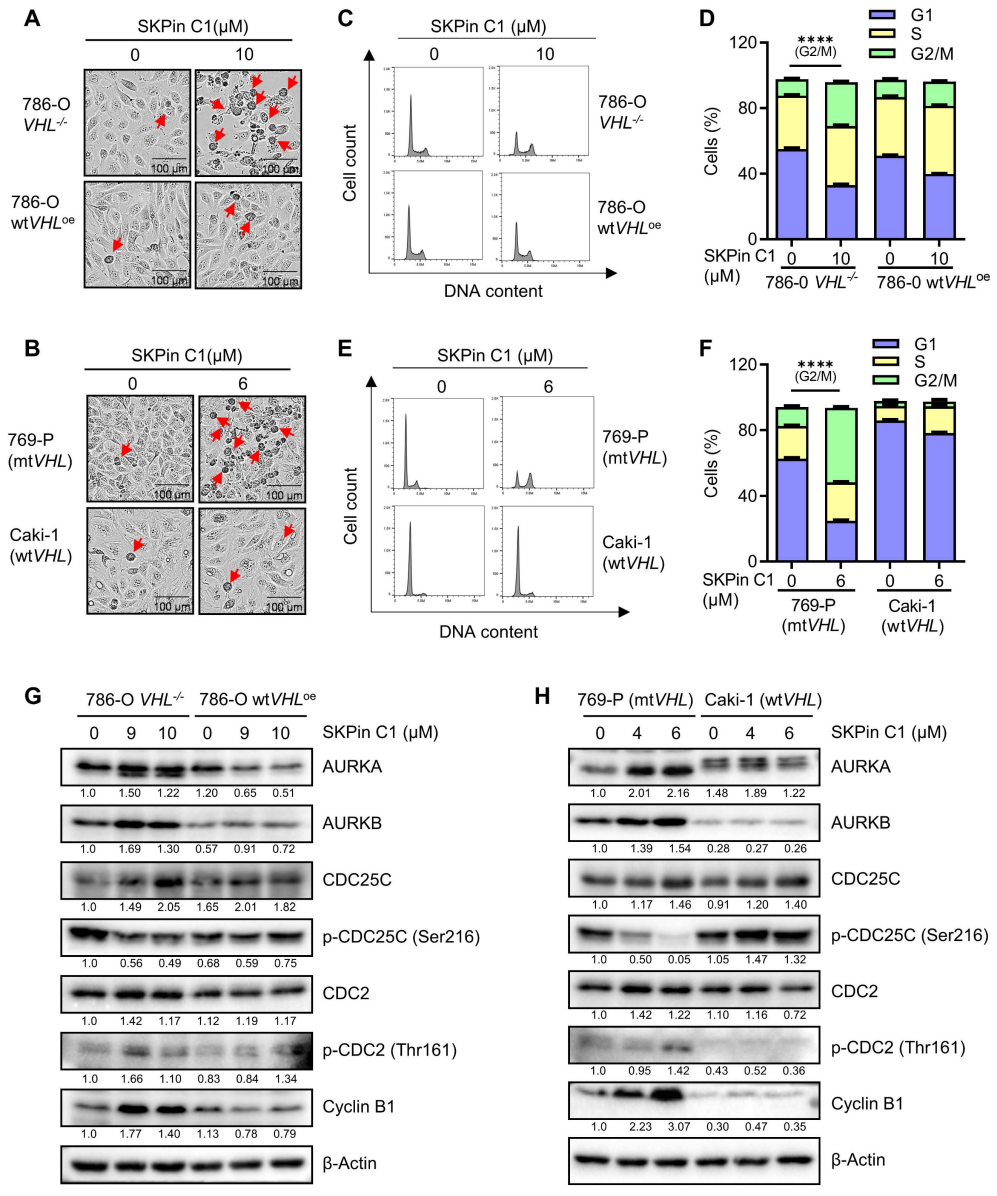
** SKPin C1 increased mitotic cell population and up-regulated mitotic kinases and cyclins in VHL-deficient RCC cells. A-B** The images of cell morphology change after SKPin C1 treatment. VHL-isogenic RCC cell pairs were treated with 10 μM SKPin C1 for 24 hours (A), and VHL non-isogenic RCC cell pairs were treated with 6 μM SKPin C1 for 24 hours (B), the representative images of cell morphology were taken by IncuCyte ZOOM. The arrow indicates the rounding cell. **C-F** SKPin C1 effect on the cell cycle. VHL-isogenic RCC cell pair (C) and VHL non-isogenic RCC cell pair (E) were treated with the indicated concentration of SKPin C1. After staining with propidium iodide (PI), the cell cycle was detected by flow cytometry. The percentage of VHL-isogenic RCC cell pair (D) and VHL non-isogenic RCC cell pair (F) distributed in the G1, S, and G2/M phases from the flow cytometry analysis. Data are shown as the mean ± SD, n=3. ****P<0.0001 between two indicated groups, Student's t-test. **G-H** Western blot analysis of cell cycle kinase and cyclin protein levels in SKPin C1-treated VHL-isogenic RCC cell pair (G) and VHL non-isogenic RCC cell pair (H).

To identify the biological target responsible for the synthetic lethal effect of SKPin C1, we analyzed various cellular phenotypes in RCC cells treated with the compound. The most striking phenotypes of SKPin C1 that differentially appeared in VHL wildtype and mutant cells were cell morphological change and cell cycle distribution. SKPin C1 induced a rounding cell morphology (while adherent to the bottom) in VHL-deficient RCC cells, which was similar to a typical mitotic cell morphology (Fig. 2A-B). Wildtype VHL-expressing cells did not show this kind of morphological change upon SKPin C1 treatment. Cell cycle analysis showed that SKPin C1 reduced G1 population but significantly increased G2/M population in VHL-deficient RCC cells (Fig. 2C-F). Based on these phenotypes, we analyzed the changes in cell cycle and mitosis-related proteins in RCC cells treated with SKPin C1. The levels of most of G1, S and G2 cyclins, including cyclins D, E, and A were not changed by SKPin C1 treatment in both VHL-wildtype and deficient RCC cells (Supplementary Fig. S3A-B). CDK4, CDK6 and p53 levels also were not changed in both cells treated with SKPin C1. Interestingly, majority of mitotic kinases and mitosis-related cyclins were significantly changed by SKPin C1 in VHL-deficient RCC cells (Fig. 2G-H). The levels of mitotic kinases AURKA and AURKB were increased in VHL-deficient RCC cells treated with SKPin C1, while they were not changed in VHL-expressing cells (Fig. 2G-H). The level of phosphorylated CDC25C at Ser216 (inhibitory phosphorylation) was dose-dependently reduced, while the level of its downstream CDK1 phosphorylation at Thr161 (activating phosphorylation) was increased by SKPin C1 in VHL-deficient RCC cells. The level of cyclin B, a mitotic cyclin and a partner of CDK1, was significantly increased by SKPin C1 in VHL-deficient RCC cells (Fig. 2G-H). These data suggested that SKPin C1 increased the population of mitotic cells in VHL-deficient RCC cells and AURKA/B-CDC25C-CDK1/cyclin B pathway was persistently up-regulated in VHL-deficient RCC cells upon treatment with SKPin C1.

### 3.3. SKPin C1 induced mitotic arrest in VHL-deficient RCC cells via disrupting spindle formation and microtubule networks

**Figure 3:**
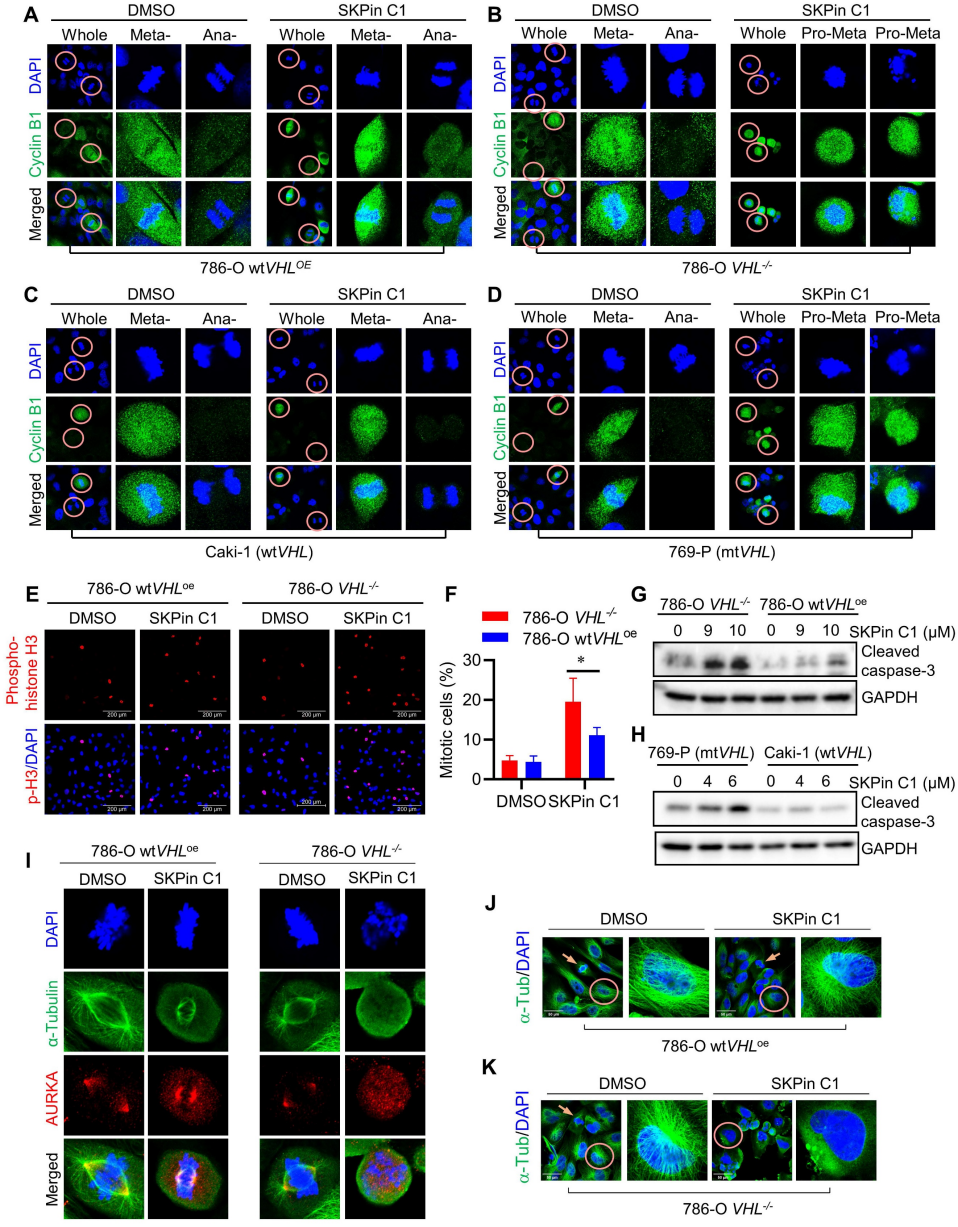
** SKPin C1 induced mitotic arrest in VHL-deficient RCC cells via disrupting spindle formation and microtubule networks. A-D** SKPin C1 effect on the expression and localization of Cyclin B. RCC cells were treated with 5 μM SKPin C1 for 24 hours. Immunofluorescence analysis of Cyclin B (green) and DNA (DAPI, blue) in SKPin C1-treated 786-O *VHL*^-/-^ cells (A), 786-O wt*VHL*^oe^ cells (B), 769-P cells (C), Caki cells (D). Enlarged images showed the representative cells in metaphase, pro-metaphase and anaphase. **E-F** Immunofluorescent analysis of Phospho-Histone H3 (Mitotic Marker). VHL-isogenic RCC cell pair were treated with 5 μM SKPin C1 for 24 hours, subsequently stained with phospho-Histone H3 antibody (red) and DAPI (blue) to show the cells undergoing mitosis (E). Quantification of the percentage of mitotic cells, the total cells are defined by the DAPI (blue) staining. Data are shown as the mean ± SD, n=3, independent experiments. For each experiment, at least 100 cells from each treatment condition were analyzed), *P<0.05 between two indicated groups, Student's t-test (F). **G-H** Western blot analysis of cleaved caspase-3 in SKPin C1-treated VHL-isogenic RCC cell pair (G) and VHL non-isogenic RCC cell pair (H). **I** Immunofluorescent analysis of mitotic spindle. VHL-isogenic RCC cell pair were treated with 5 μM SKPin C1 for 24 hours, subsequently stained with α-tubulin antibody (green), Aurora A (red), and DAPI (blue) to show the mitotic spindle morphology. Aurora A localizes at the centrosome in metaphase to present the spindle pole. **J-K** Immunofluorescent analysis of microtubule network. 786-O wt*VHL*^oe^ cells (J),786-O *VHL*^-/-^ (K) were treated with 5 μM SKPin C1 for 24 hours, subsequently stained with α-tubulin antibody (green) and DAPI (blue). Enlarged images showed the representative microtubule. The arrow showed the mitotic cells.

To explore mitotic phenotypes of VHL-deficient RCC cells treated with SKPin C1 in greater detail, we analyzed cyclin B expression in RCC cells using immunofluorescent labeling. Cyclin B is a mitotic cyclin whose expression reaches the highest-level during mitosis. It forms a complex with CDK1 and plays a critical role in mitotic progression to metaphase. After successful metaphase alignment of sister chromatids on the spindle, cyclin B/CDK1 complex activates the anaphase promoting complex/cyclosome (APC/C), which in turn stimulates the destruction of cyclin B and securin, leading to anaphase transition and mitotic exit^27^. Therefore, cyclin B protein level is reduced sharply during the metaphase-anaphase transition. In 786-O wt*VHL*^oe^ cells, a high cyclin B level was observed in metaphase cells, while very little level was observed in anaphase cells (Fig. 3A). SKPin C1 treatment did not change this pattern in the VHL-expressing cells. However, in 786-O *VHL^-/-^* cells, SKPin C1 treatment results in a high level of cyclin B in the majority of cells (Fig. 3B, Fig. S4A).

Most cells were synchronized in pro-metaphase, and no anaphase cells, there is a significant increasement of cyclin B-positive cells were observed in 786-O *VHL^-/-^* cells treated with SKPin C1. A very similar cell synchronization phenotype was observed in VHL non-isogenic RCC cell pair treated with SKPin C1 (Fig. 3C-D, Fig. S4B). To verify whether the cells were synchronized in mitotic phase, we labeled the cells with phospho-histone H3 (Ser 10), a specific nuclear marker for cells undergoing mitosis^28^, ^29^. The results showed that there are significantly more mitotic cells in 786-O *VHL^-/-^* cells treated with SKPin C1 than in 786-O wt*VHL*^oe^ cells treated with the compound (Fig. 2E-F). To examine whether the increase in the number of mitotic cells by the compound was due to the promotion of mitotic entry or induction of mitotic arrest and death, we analyzed an apoptosis marker (caspase-3 cleavage) in RCC cells treated with SKPin C1. SKPin C1 dose-dependently increased the level of cleaved caspase 3 in VHL-deficient RCC cells, implying that the compound induced mitotic arrest and death in the cells (Fig. 3G-H). To further analyze mitotic phenotypes of cells treated with SKPin C1, we observed mitotic spindle and centrosomes using α-tubulin and AURKA immunofluorescent labeling. SKPin C1 treatment slightly shortened the spindle length in 786-O wt*VHL*^oe^ cells, but the cells underwent mitosis progression with normal centrosomal distribution of AURKA (Fig. 3I). However, it completely disrupted spindle formation in *VHL^-/-^* cells and caused a significant increasement of abnormal spindle/chromosome (Fig. 3I, Fig. S4C). Moreover, high AURKA level was observed throughout the nucleus without centrosomal localization in these cells. These results suggested that cyclin B/CDK1 pathway was activated and AURKA level was increased normally during G2/M transition and early mitotic entry steps in VHL-deficient cells treated with SKPin C1. However, the step of spindle formation and centrosome segregation was completely disrupted by the compound, leading to mitotic frozen of the cells at the pro-metaphase and accumulation of cyclin B and AURKA levels. We hence suspected that the mitotic phenotypes induced by SKPin C1, i.e. defective spindle formation and centrosome segregation, in VHL-deficient cells was attributable to the possible defect in microtubule structure or assembly. We then analyzed microtubule networks in mitotic and non-mitotic RCC cells treated with SKPin C1. SKPin C1 slightly reduced microtubule network intensity in 786-O wt*VHL*^oe^ cells, while the cells underwent normal mitosis (Fig. 3J). In 786-O *VHL^-/-^* cells, SKPin C1 largely disrupted microtubule networks in non-mitotic cells and there were no normal mitotic cells observed (Fig. 3K). Similar mitotic phenotypes were observed in VHL non-isogenic RCC cell pair treated with SKPin C1 (Supplementary Fig. S4D-F). These data demonstrated that SKPin C1 induced mitotic arrest in VHL-deficient RCC cells at the pro-metaphase via disrupting spindle formation and microtubule networks. Again, silencing of SKP2 had no effect on microtubule network formation in either VHL-deficient or expressing cells, further confirming that SKP2 was not the target protein responsible for the SKPin C1 effect on mitotic phenotypes (Supplementary Fig. S4G).

### 3.4. SKPin C1 binds to tubulin and destabilizes microtubule

**Figure 4:**
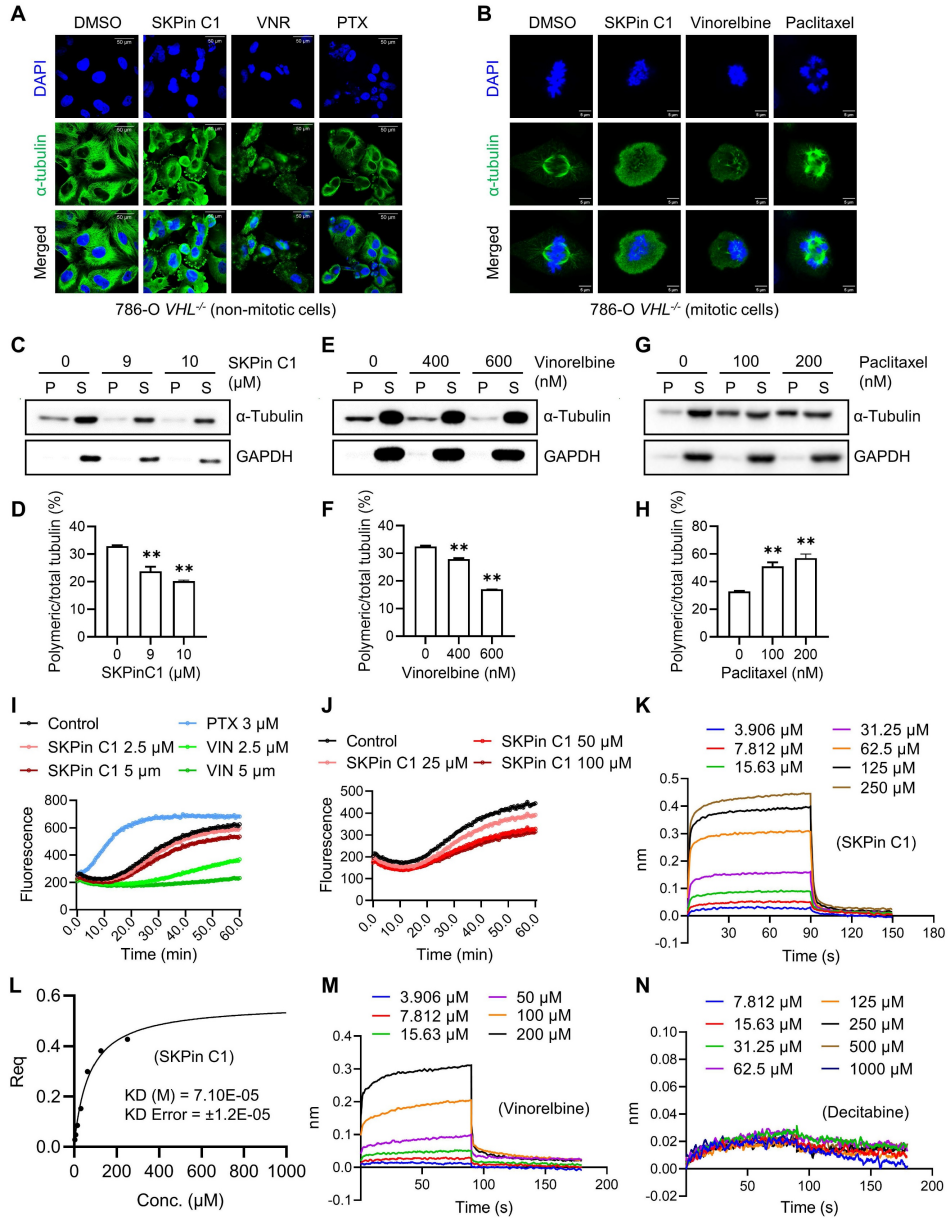
** SKPin C1 binds to tubulin and destabilizes microtubule. A-B** Comparison the effect of SKPin C1 with representative microtubule targeting agents on microtubule network and spindle formation. 786-O *VHL*^-/-^ cells were treated with 5 μM SKPin C1, 400nM Vinorelbine, 200nM Paclitaxel for 24 hours, stained with α-tubulin antibody(green) and DAPI (blue) to monitor the cell microtubules (A) and mitotic spindle morphology (B). **C-H** Evaluation of the effect of drugs on microtubule polymerization in a cell population. 786-O *VHL*^-/-^ cells were treated with the indicated concentration of SKPin C1 (C), vinorelbine(E), or paclitaxel(G) for 24 hours, the α-tubulin protein in soluble fractions (S) and polymerized fractions (P) were analyzed by western blotting, GAPDH was used as loading control. Quantification of the ratio of the polymerized/total α-tubulin (sum of the soluble and polymerized fractions) with the treatment of SKPin C1 (D), vinorelbine (F), or paclitaxel (H). Data are shown as mean ± SD (n=3). **P < 0. 01 between two indicated groups, one-way ANOVA (D, F, H). **I-J** Tubulin polymerization assay in vitro performed to determine drugs effect on tubulin polymerization activity with the indicated concentration. Paclitaxel was used as a positive control, Vinorelbine was used as a negative control. **K-N** Biolayer interferometry (BLI) analysis of the interaction between drugs and tubulin protein. Biotin-labeled tubulin was dipped in increasing concentration of SKPin C1 (K), vinorelbine (M) or decitabine (N) to measure their binding affinity with tubulin. L Steady state graph showed the KD (M) value between SKPin C1 and tubulin protein.

As we observed that SKPin C1 disrupted the microtubule network in VHL-deficient RCC cells, and this seemed to be the primary cause of the compound to induce mitotic arrest, we investigated the effect of SKPin C1 on microtubule at the molecular level. We first analyzed the microtubule network and mitotic phenotypes of SKPin C1 in comparison with those of known anti-microtubule agents, including the microtubule destabilizer vinorelbine and the microtubule stabilizer paclitaxel^30^. SKPin C1 effect on microtubule network was more similar to that of vinorelbine, which caused weaken and disrupted microtubule networks in non-mitotic VHL-deficient cells (Fig. 4A). Moreover, in mitotic cells, SKPin C1 and vinorelbine disrupted spindle poles with almost no detectable spindle fibers, while paclitaxel caused the formation of thick, misaligned spindles with multiple spindle poles (Fig. 4B). We next conducted a cell-based microtubule polymerization assay by fractionating cells into polymeric microtubule fraction (containing polymerized microtubules) and soluble fraction (containing non-polymeric, free tubulins) and quantitated the effect of SKPin C1 on microtubule polymerization in 786-O *VHL^-/-^* cells. As a result, SKPin C1 and vinorelbine significantly reduced the level of polymeric microtubules in the cells, while paclitaxel increased it (Fig. 4C-H). These results suggested that SKPin C1 destabilized microtubules in a manner similar to vinorelbine. Next, we performed an in vitro tubulin polymerization assay with an assay kit containing purified tubulin protein and fluorescent reporter chemical that is incorporated into tubulin polymers during the polymerization reaction. SKPin C1 weakly but dose-dependently inhibited tubulin polymerization in vitro (Fig. 4I). Paclitaxel highly increased the tubulin polymerization rate, while vinorelbine almost completely inhibited the polymerization.

Higher concentrations of SKPin C1 further inhibited the tubulin polymerization, but, unlike vinorelbine, it did not completely inhibit it (Fig. 4J). Lastly, we conducted a molecular interaction assay for SKPin C1 and tubulin protein using the Biolayer Interferometry (BLI) assay where biotinylated-tubulin was immobilized onto a streptavidin-coated biosensor. The BLI result showed that SKPin C1 directly bound to tubulin protein with an estimated *K_D_* value of 71 μM (Fig. 4K-L). Vinorelbine was used as a positive control and decitabine, a DNA methyltransferase inhibitor that is unrelated to microtubule, was used as a negative control for tubulin binding (Fig. 4M-N). Collectively, these results indicate that SKPin C1 binds to tubulin and acts as a microtubule destabilizing agent. Our data further suggest that microtubule dynamics could be a relevant target of SKPin C1 for its abnormal mitotic phenotypes in VHL-deficient RCC cells.

### 3.5. Microtubule dynamics and stability are altered in VHL-deficient RCC cells

**Figure 5:**
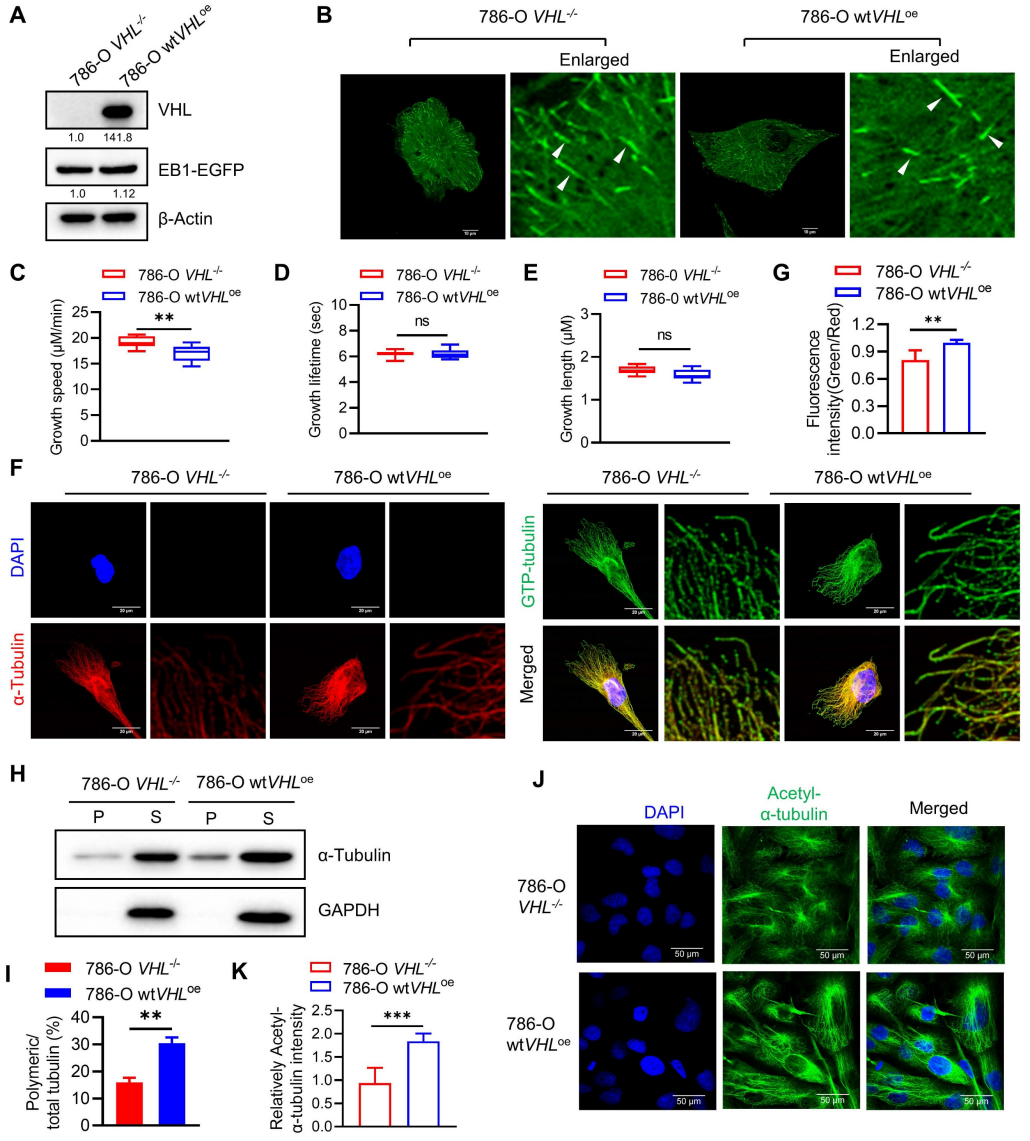
** Microtubule dynamics and stability are altered in VHL-deficient RCC cells. A-B** Overexpression of EB1-EGFP in VHL-isogenic RCC cell pair. Western blot analysis of EB1-EGFP expression (A). EB1-EGFP comets were taken by Nikon confocal A1 and indicated with the white arrows (B). **C-E** Evaluation of microtubule dynamics in VHL-isogenic RCC cell pair. The EB1-EGFP videos were recorded by a Nikon TiE Widefield Microscope. Parameters of microtubule dynamics were analyzed with the plusTip Tracker, including growth speed (C), growth lifetime (D), and growth length (E). Data are shown as the mean ± SD, n=5 independent experiments. For each experiment, at least 9 cells from each treatment condition were analyzed. **P < 0.01, Student's t-test. ns denotes not significant. **F-G** Analysis of the GTP-tubulin amount in VHL-isogenic RCC cell pair. Immunofluorescence staining for GTP-tubulin (green), α-tubulin (red) and DAPI (blue), the fluorescence intensity in the green and red channels was quantified using image J, followed by calculating the fluorescence intensity ratio (green/red). Data are shown as the mean ± SD, n=5, **P < 0.01 between two indicated groups, Student's t-test (G). **H-I** Detection of the polymerized tubulin amount in VHL-isogenic RCC cell pair. The α-tubulin protein in soluble fractions (S) and polymerized fractions (P) were analyzed by western blotting, GAPDH was used as loading control (H). Quantification of the ratio of the polymerized/total α-tubulin (sum of the soluble and polymerized fractions). Data are shown as mean ± SD (n=3). **P < 0.01 between two indicated groups, Student's t-test (I). **J-K** Comparation of the acetyl-α-Tubulin in VHL-isogenic RCC cell pair via immunofluorescence staining (J), and the immunofluorescence intensity of acetyl-α-Tubulin were quantified, data are shown as mean ± SD. ***P<0.001 between two indicated groups, Student's t-test (K).

We next explored potential mechanisms underlying the hypersensitivity of VHL-deficient cells to microtubule destabilizing agents. It has been reported that VHL is a microtubule-associated protein that could protect microtubules from depolymerization^31^. To verify the effect of VHL deficiency on the microtubule stability in RCC cell lines, we first analyzed the microtubule growth dynamics in VHL-isogenic RCC cells using live-cell EB1 imaging analysis. EB1 is a plus-end-tracking protein (+TIP) that accumulates at growing microtubule ends^32^, and thus it can show the growth and shrinkage of microtubules in live cells. EB1-EGFP plasmid was stably expressed in 786-O VHL-isogenic cells and microtubule growth dynamics was analyzed with the time-lapse live-cell imaging of EB1 fluorescence (Fig. 5A-B). Microtubule growth lifetime and length were similar between 786-O wt*VHL*^oe^ and *VHL^-/-^* cells, but microtubule growth speed was increased in 786-O *VHL^-/-^* cells (Fig. 5C-E). We next analyzed the microtubule stability difference between 786-O wt*VHL*^oe^ and *VHL^-/-^* cells using the GTP-tubulin immunofluorescence. GTP-to-GDP transition on the microtubule end is a crucial determinant for microtubule growth and catastrophe^33^. GTP-tubulin cap at the growing end of the microtubule can facilitate microtubule polymerization and stability^34^. Immunofluorescence staining of GTP-tubulin and total tubulin showed that the intensity ratio of GTP-tubulin to total tubulin was higher in 786-O wt*VHL*^oe^ cells than in *VHL^-/-^* cells (Fig. 5F-G). We further analyzed the difference in microtubule stability between the two VHL-isogenic cell lines using in-cell microtubule polymerization assay and acetylated-tubulin staining^35^. Our results showed that VHL-deficient RCC cells have significantly reduced polymeric microtubules (Fig. 5H-I) and acetylated-tubulin level (Fig. 5J-K). These data indicate that VHL deficiency increases microtubule growth speed, but the stability of polymeric microtubule is significantly weakened in VHL-deficient RCC cells.

### 3.6. SKPin C1 sensitizes microtubule dynamics in VHL-deficient RCC

**Figure 6:**
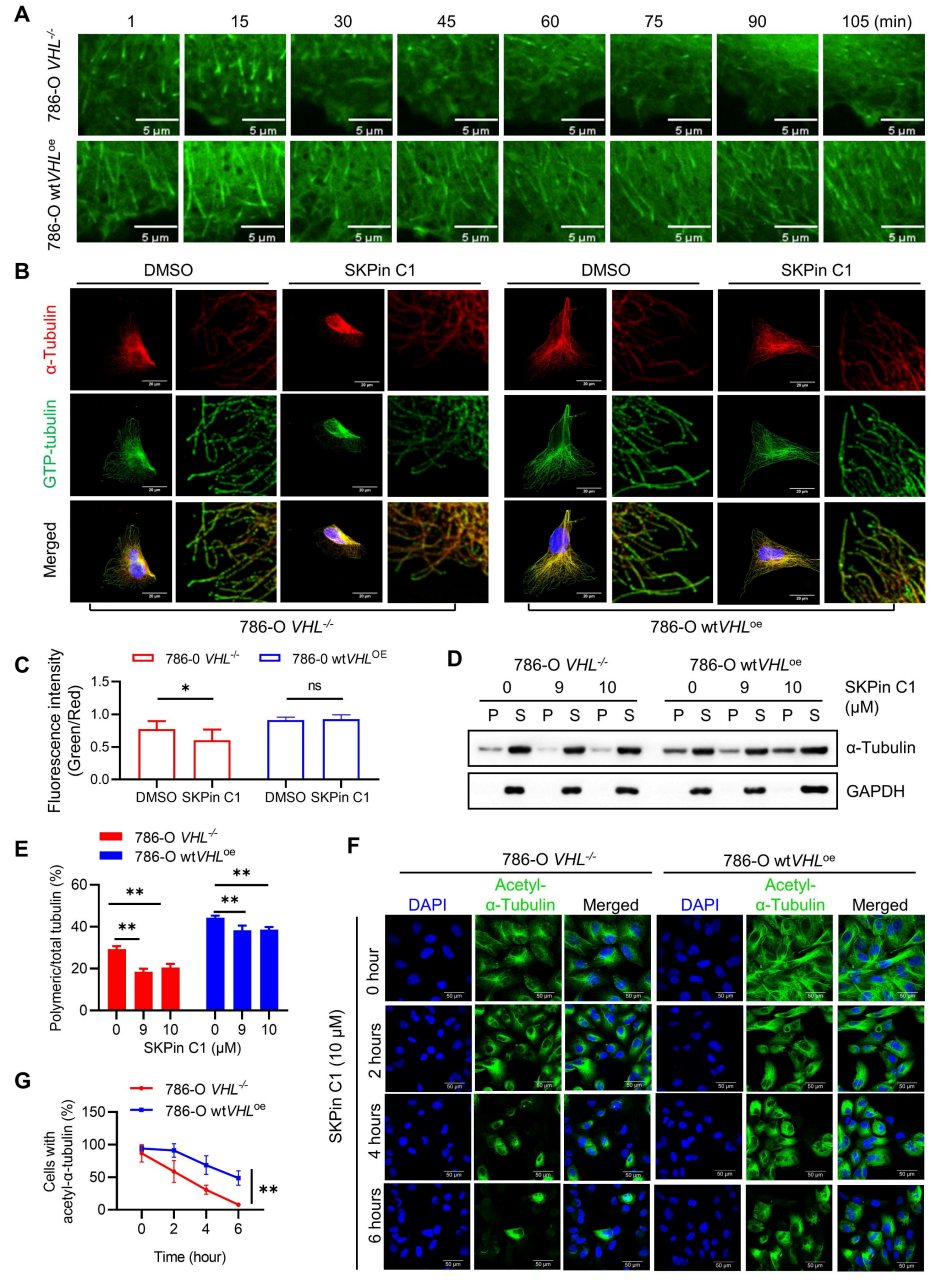
** SKPin C1 sensitizes microtubule dynamics in VHL-deficient RCC. A** SKPin C1 effect on microtubule dynamics in VHL-isogenic RCC cell pairs. Single frame of the video acquired from Olympus SpinSR10 Spinning Disk Confocal Microscope at different time points. **B-C** VHL-isogenic RCC cell pair were treated with 5 μM SKPin C1 for 24 hours, representative immunofluorescence staining for GTP-tubulin (green) and α-tubulin (red) (B), and the immunofluorescence intensity were quantified, data are shown as mean ± SD. *P<0.05 between two indicated groups, Student's t-test. ns denotes not significant (C). **D-E** VHL-isogenic cell pair were treated with 9 μM,10 μM SKPin C1 for 24 hours, the α-tubulin protein in soluble fractions (S) and polymerized fractions (P) were analyzed by western blotting, GAPDH was used as loading control (D). Quantification of the ratio of the polymerized/total α-tubulin (sum of the soluble and polymerized fractions). Data are shown as mean ± SD (n=3). **P<0.01 between two indicated groups, Student's t-test (E). **F-G** VHL-isogenic cell pair were treated with 10 μM SKPin C1 for different times and subsequently stained with Acetyl-α-Tubulin antibody (green) and DAPI (blue) to monitor the stabilized microtubule changes (F). Quantification of the ratio of cells with Acetyl-α-Tubulin and the total number of the cells which defined by DAPI (blue) staining. Data are shown as mean ± SD (n=3), **P<0.01 between two indicated groups, Two-way ANOVA (G).

Since we observed that VHL deficiency significantly decreased microtubule stability in RCC cells, we wondered whether SKPin C1 sensitizes the altered microtubule dynamics in VHL-deficient RCC cells. We first evaluated the effect of SKPin C1 on microtubule dynamics using the EB1 live-cell imaging analysis. EB1-GFP expressing cells were treated with SKPin C1 and the EB1 live-cell fluorescence was observed for 105 min under a Spinning Disk Confocal Microscope. In 786-O *VHL^-/-^* cells treated with SKPin C1, the EB1 comets became dim rapidly within an hour, while the compound did not affect the EB1 comets until the end of the tracking experiment (105 min) in 786-O wt*VHL*^oe^ cells (Fig. 6A), demonstrating that SKPin C1 rapidly shut down microtubule growth in VHL-deficient RCC cells. Moreover, SKPin C1 significantly reduced the ratio of GTP-tubulin to total tubulin in VHL-deficient cells compared to VHL-expressing ones (Fig 6B-C). In addition, the level of polymeric microtubule was significantly decreased in SKPin C1-treated 786-O *VHL^-/-^* cells, but was marginally reduced in 786-O wt*VHL*^oe^ cells treated with the compound (Fig. 6D-E). Lastly, SKPin C1 almost completely reduced the level of acetylated tubulin within 6-hour treatment in 786-O *VHL^-/-^* cells, while 786-O wt*VHL*^oe^ cells exhibited a decent level of acetylated tubulin at the same treatment condition (Fig. 6F-G). These results demonstrate that VHL-deficient RCC cells have altered microtubule dynamics and SKPin C1 sensitizes the altered microtubule dynamics in the cells, leading to a significant microtubule destabilization.

### 3.7. VHL loss is synthetic lethal with anti-microtubule agents in RCC cells

**Figure 7:**
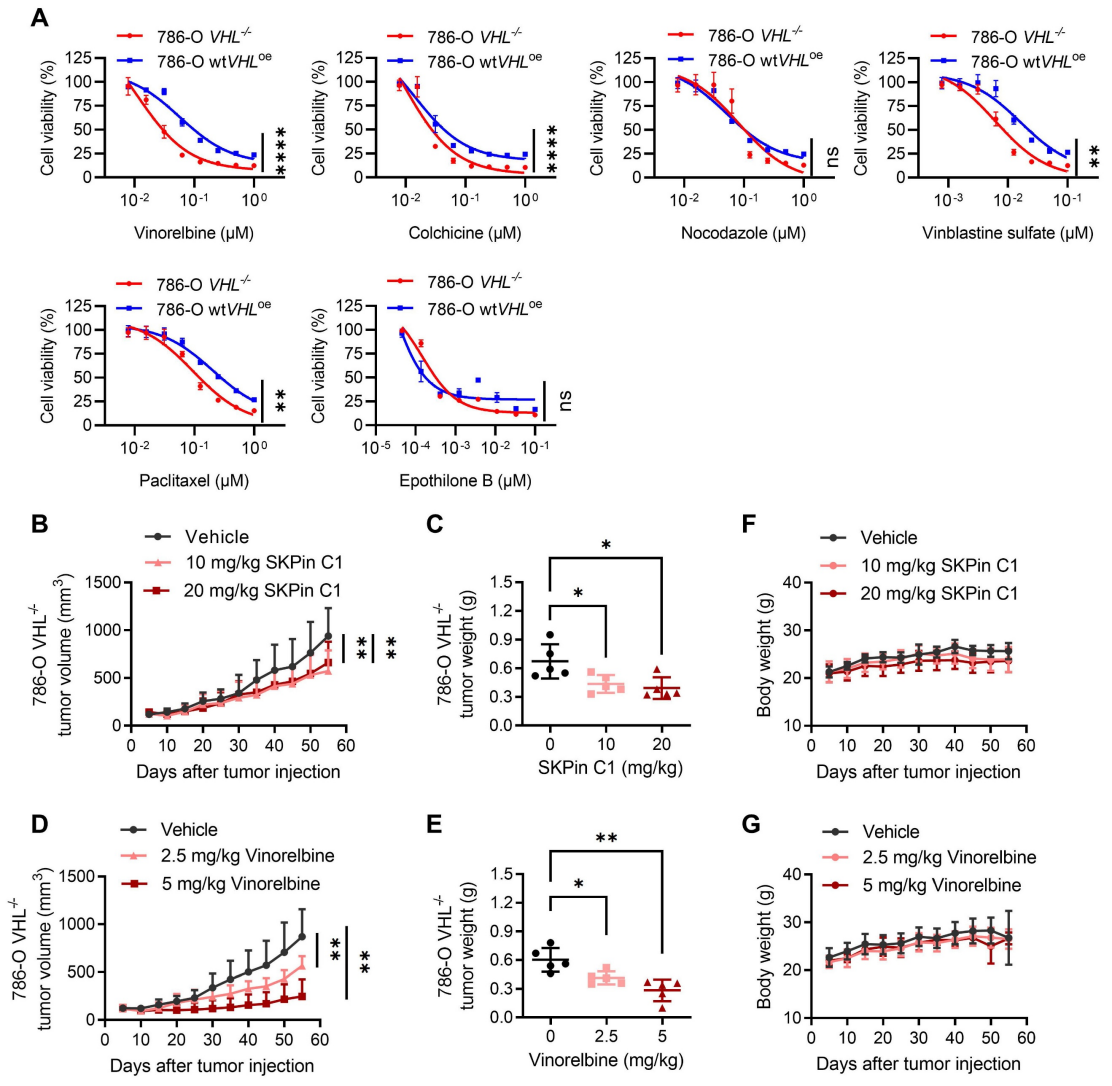
** VHL loss is synthetic lethal with anti-microtubule agents in RCC cells. A** Dose-response curve of 786-O VHL-isogenic cell pair treated with different microtubule targeting agents for 72 hours. Data are shown as the mean ± SD, n=3. ****P<0.0001, **P<0.01 between two indicated groups, two-way ANOVA. ns denotes not significant. **B-G** SKPin C1, vinorelbine inhibited tumor growth in the 786-O cell xenograft mice model. Tumor growth curve analysis of 786-O cell xenograft mice treated with SKPin C1 (B) or vinorelbine (D). Data are shown as the mean ± SD, n=5, **P < 0.01, between two indicated groups, one-way ANOVA. Tumor wet weight of 786-O cell xenograft mice treated with SKPin C1 (C) or vinorelbine (E). Data are shown as the mean ± SD, n=5, *P < 0.05 between two indicated groups, two-way ANOVA. Body weight analysis of 786-O cell xenograft mice treated with SKPin C1 (F) or vinorelbine (G). Data are shown as the mean ± SD, n=5.

To verify whether the synthetic lethality of VHL and SKPin C1 is mediated by the disruption of microtubule dynamics, we examined various anti-microtubule agents with different classes, including microtubule destabilizing agents such as vinorelbine, vinblastine, colchicine and nocodazole, and microtubule stabilizing agents such as paclitaxel and epothilone B. Results showed that 786-O *VHL^-/-^* cells were generally more sensitive to anti-microtubule agents than 786-O wt*VHL*^oe^ cells (Fig. 7A), but the synthetic lethal effect varies depending on the class of agents. Among the tested anti-microtubule agents, vinorelbine and vinblastine, both vinca alkaloid class drugs, showed the highest differential sensitivity between 786-O *VHL^-/-^* and wt*VHL*^oe^ cells. These data imply that anti-microtubule agents can induce synthetic lethality in VHL-deficient RCC cells, but the synthetic lethal effect may vary depending on microtubule binding site and inhibitory mechanism. We also analyzed a docking simulation to explore a potential binding site of SKPin C1 on microtubules and found that SKPin C1 fits well into the β-/α-tubulin interface, similar to the colchicine binding site (Supplementary Fig. S5A-F). The precise binding site and inhibition mechanism of SKPin C1 on microtubule need to be further elaborated with crystal structure analysis of microtubule-drug complex.

Next, to evaluate the synthetic lethal effect of anti-microtubule agents in vivo, we performed mouse tumor xenograft experiments for SKPin C1 and vinorelbine with VHL-deficient 786-O cells. Both SKPin C1 and vinorelbine significantly inhibited the tumor growth of 786-O *VHL^-/-^* xenografts in nude mice (Fig. 7B-E). Both drugs did not show reduction in mice body weights (Fig. 7F-G), suggesting that there was no apparent toxicity to the mice at the indicated doses. We noticed that there was no further reduction in tumor size at the higher dose of SKPin C1 (10 mg/kg vs 20 mg/kg), and vinorelbine showed much stronger antitumor effect than SKPin C1 on VHL-deficient RCC tumor xenograft. It is possibly due to the poor solubility and limited bioavailability of SKPin C1 in the in vivo condition. Since SKPin C1 was originally designed to inhibit the binding interface between SKP2 and p27, it is necessary to optimize the compound for better fit into microtubule and in vivo bioavailability to develop more potent leads for VHL synthetic lethal drug.

### 3.8. Combination of anti-microtubule agent and HIF-2α inhibitor enhanced the antitumor effect on VHL-deficient RCC cells

**Figure 8:**
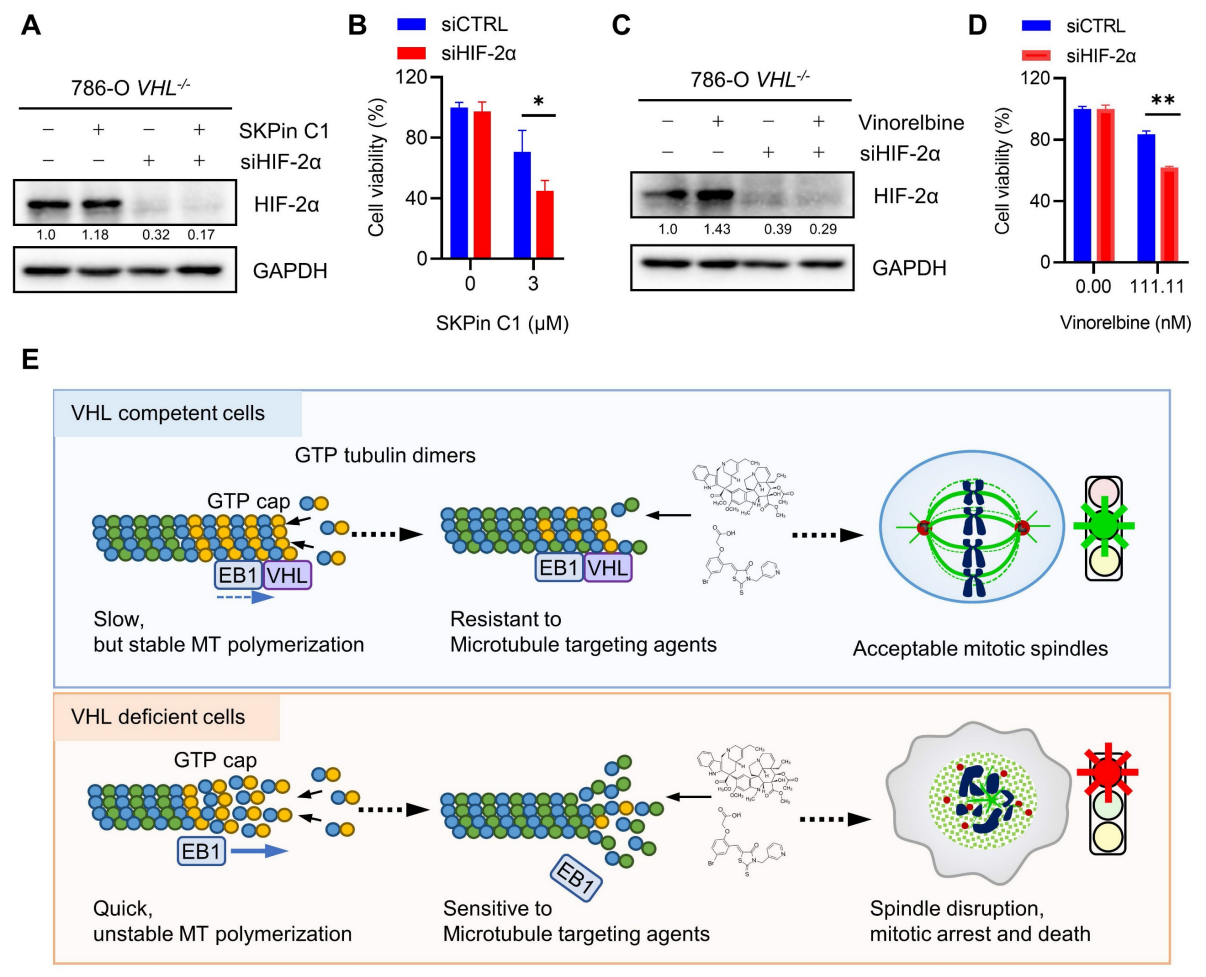
** Combination of anti-microtubule agent and HIF-2α inhibitor enhanced the antitumor effect on VHL-deficient RCC cells. A-D** Cotreatment of SKPin C1 (B) or vinorelbine (D) with HIF-2α siRNA in 786-O cells for 72 hours, the cell viability was detected by alarm blue assays. Data are shown as the mean ± SD, n=3, *P<0.5, **P<0.01 between two indicated groups, Student's t-test. Western blot analysis of the expression of HIF-2α (A, C). **E** Proposed working model of the synthetic lethality between microtubule dynamic disruption and VHL deficiency.

It has been reported that metastatic and advanced RCC are generally resistant to chemotherapy drugs. Anti-microtubule drugs, such as vinblastine and docetaxel, when applied as single agents or combined with conventional immunotherapy drugs, also produced very limited antitumor efficacy in advanced RCC^36^. However, the precise mechanism of the RCC resistance to chemotherapy drugs has not been understood yet, implying a concern for applying our findings in the clinical setting. Giannakakou and coworkers reported interesting observations that microtubules regulate trafficking and activity of HIF-α, and anti-microtubule drugs can reduce the level and activity of HIF-α in cancer cells^37^. However, the connection between microtubule and HIF-α regulation was not present in RCC cells^38^. Therefore, it was postulated that the drug resistance of metastatic RCC to anti-microtubule drugs could be due to HIF-α accumulation following the drug treatment. We then tested SKPin C1 and vinorelbine in combination with HIF-α inhibitors, including HIF-2α siRNA and a small molecule inhibitor belzutifan, which binds to HIF-2α and prevents the complex formation with HIF-1β^39^. As expected, both SKPin C1 and vinorelbine could not reduce the level of HIF-2α in VHL-deficient RCC cells (Fig. 8A and C). The silencing of HIF-2α using specific siRNA significantly enhanced the sensitivity of 786-O *VHL^-/-^* cells to both SKPin C1 and vinorelbine (Fig. 8A-D). Moreover, the HIF-2α inhibitor belzutifan also enhanced the vinorelbine sensitivity on 786-O *VHL^-/-^* cells (Supplementary Fig. S6A-B). However, belzutifan could not enhance SKPin C1's effect on VHL-deficient RCC cells (Supplementary Fig. S6C), implying that there could be a varying combination effect of HIF-2α specific inhibitor with different classes of anti-microtubule agents. Taken together, our study shows that VHL-deficient RCC cells are synthetic lethal with anti-microtubule agents via disrupting mitotic progression and provides a rationale for potential drug combination with newly developed RCC targeted therapies, such as HIF inhibitors.

## 4. Discussion

VHL is the most frequently mutated or inactivated tumor suppressor in RCC, with over 50% of RCC patients exhibiting a loss of VHL, making it a crucial biomarker for the discovery of therapeutic vulnerability in RCC. In this study, we report that SKPin C1, a small molecule inhibitor of the E3 ubiquitin ligase SKP2, induces synthetic lethality in VHL-deficient RCC cells. In our detailed mechanistic study, we discovered that the synthetic lethal effect of SKPin C1 in VHL-deficient RCC cells was due to its impact on microtubule dynamics, rather than SKP2 inhibition. We provided a series of evidence to support our model of the synthetic lethality: (1) SKPin C1 selectively induces mitotic arrest by disrupting microtubule networks in VHL-deficient cells. (2) SKPin C1 binds to tubulin and inhibits tubulin polymerization in vitro and in cells. (3) VHL contributes to microtubule dynamics, and VHL loss reduces microtubule stability. (4) VHL-deficient RCC cells are hypersensitive to anti-microtubule agents, including the clinical drug vinorelbine. These results were further validated in mouse xenograft tumor model. This study suggests that the microtubule dynamics is a therapeutic vulnerability in VHL-deficient RCC.

SKPin C1 is a small-molecule inhibitor of SKP2-mediated p27 degradation. SKP2 (S-phase kinase-associated protein 2) is a key component of the SKP2-SCF E3 ubiquitin ligase complex where it acts as the substrate recognition factor^40^, ^41^. SKP2 is involved in the ubiquitin-mediated degradation of several key cell cycle regulators, including p27 (CDKN1B)^42^. SKPin C1 was designed to impede the binding between SKP2 and p27, thus preventing SKP2-mediated degradation of p27^25^. As p27 is an inhibitor of cyclin D/CDK4 or cyclin E/CDK2, the accumulation of p27 upon SKPin C1 treatment leads to the blockade of G1/S transition. Indeed, SKPin C1 has been observed to block G1/S transition in a p27-dependent manner in several cancer types^26^, ^43^. Since we initially found SKPin C1 as an inducer of synthetic lethality in VHL-deficient RCC cells, we sought to determine the causal relationship between SKP2 inhibition and VHL loss in the cells. However, SKPin C1 strongly induced G2/M cell cycle arrest in VHL-deficient cells, and SKP2 depletion had no effect on the cell viability in VHL-deficient RCC cells. Furthermore, SKPin C1 disrupted microtubule networks in VHL-deficient cells, causing spindle deformation and mitotic arrest, while SKP2 depletion had no effect on these phenotypes. These data suggested that the synthetic lethal effect of SKPin C1 was due to its off-target effect. We therefore attempted to identify the causative target protein of SKPin C1 for its synthetic lethal effect with VHL loss. Our detailed biochemical and phenotypic studies revealed that SKPin C1's synthetic lethal effect was primarily due to its anti-microtubule activity. SKPin C1 directly bound to tubulin and inhibited microtubule polymerization. We also showed that VHL loss altered microtubule dynamics, i.e. promoting microtubule growth speed while reducing microtubule stability. SKPin C1 treatment strongly reduced microtubule stability, followed by the disruption of mitotic spindles in VHL-deficient RCC cells, making the cells hypersensitive to the compound. This phenomenon was also observed with clinical anti-microtubule agents, particularly with the microtubule destabilizer vinorelbine, demonstrating that microtubule is a causative target protein of SKPin C1 for its synthetic lethal effect on VHL-deficient RCC cells.

Although SKPin C1 was shown to bind and inhibit microtubule polymerization, the inhibition profile was not identical to the microtubule destabilizing agent vinorelbine. SKPin C1 could not completely inhibit tubulin polymerization in vitro, even at the concentrations 20 times higher than its IC50, while vinorelbine completely shut down the tubulin polymerization. Microtubule has many different binding sites for small molecules, and at least 4-5 different classes of anti-microtubule agents that bind to different sites within microtubule complex were known. Based on our molecular docking simulation, SKPin C1 fits well into the β-/α-tubulin interface of the microtubule, which is very similar to the colchicine binding pocket. Furthermore, SKPin C1's in vivo antitumor effect was not as strong as vinorelbine. It is presumably due to its nature of a protein-protein interaction inhibitor, which needs very precise molecular structure to impede the interaction between two huge proteins in their interface. This would limit the structural modification for better pharmacological profile and druggability. However, there should be much bigger room for structural modifications when SKPin C1 is developed for microtubule inhibitor. Further studies on its precise binding site on microtubule and the mode of interaction are needed to design better drug leads with acceptable pharmacological profile.

VHL has multifaceted roles in diverse biological processes. In addition to its canonical role in HIF-dependent pathways, VHL is also involved in transcriptional regulation^44^, senescence^45^ and the formation of the extracellular matrix^46^ as HIF-independent pathways. In particular, it was reported that VHL is associated with tubulin and regulates microtubule stability. Hergovich *et al.*, demonstrated that VHL colocalizes and binds with microtubules in cells, preventing nocodazole-induced microtubule depolymerization^31^. Thoma and colleagues demonstrated that VHL inactivation leads to destabilization of astral microtubules, resulting in spindle misorientation and chromosome instability^47^. Using a real-time EB3 tracking on microtubules, they also found that VHL could reduce the turnover rate of microtubule growth and shrinkage, thereby promoting microtubule stability^48^. These observations were further corroborated by the study conducted by Frew *et al.*, showing the HIF-independent, microtubule-regulating functions of VHL in mouse primary cells derived from VHL knockout mice^49^. Our study also showed that 786-O *VHL^-/-^* cells exhibited increased microtubule growth speed and a higher level of GTP-tubulin compared to the 786-O wt*VHL*^oe^ cells. We further showed that 786-O *VHL^-/-^* cells have significantly lower levels of acetylated microtubules and polymerized microtubules compared to those observed in 786-O wt*VHL*^oe^ cells. Treatment of VHL-deficient cells with anti-microtubule agents, including SKPin C1 and vinorelbine, significantly worsen these microtubule phenotypes, leading to the disruption of mitotic spindles and subsequent cell death (Summarized in Fig. 8E). We therefore concluded that microtubule dynamic instability in VHL-deficient cells is a therapeutic vulnerability to anti-microtubule agents.

Microtubules are one of the most important components of the cytoskeleton, providing structure and shape for eukaryotic cells, and play an important role in many cellular processes, including intracellular transport, cell migration and cell division^50^, ^51^. Given the importance of microtubules in cell division, it has become an effective target for cancer treatment^30^, ^52^. Microtubule-targeted drugs, including microtubule stabilizing agents and destabilizing agents, have achieved great success as chemotherapy drugs in a number of types of cancer the clinic. However, metastatic and advanced RCC are known to be very resistant to chemotherapy drugs, including anti-microtubule agents. In particular, the microtubule destabilizing agent vinblastine and the stabilizing agent docetaxel have been used in the clinical trials for advanced RCC, but failed to achieve meaningful clinical outcomes^36^. Although the mechanism of the resistance to anti-microtubule agents in advanced RCC has not been elucidated yet, it has been postulated that RCC cells have a distinct, microtubule-independent HIF-α nuclear transport mechanism, and thus anti-microtubule agents are unable to reduce HIF-α level in RCC cells^38^. Combining an anti-microtubule agent with a HIF-α inhibitor, such as belzutifan^39^ shown in our study, may improve the antitumor efficacy of anti-microtubule agents in advanced RCC. Moreover, personalized application of anti-microtubule agent alone or combination with a HIF-α inhibitor in VHL-mutant subtypes of RCC will be a potential precision medicine strategy for the treatment of advanced RCC.

## Supplementary Material

Supplementary figures and tables.
