# Supplementary material for: Microtubule dynamics is a therapeutic vulnerability in VHL-deficient renal cell carcinoma
Source: Int J Biol Sci. 2025 Apr 28;21(7):3286–305. doi: 10.7150/ijbs.109642 (PMC12080383; doi:10.7150/ijbs.109642)
Supplement: Supplementary file 1 — Supplementary figures and tables. [file ijbsv21p3286s1.pdf]

Microtubule dynamics is a therapeutic vulnerability in VHL-deficient renal cell carcinoma

Supplementary data

Figure S1

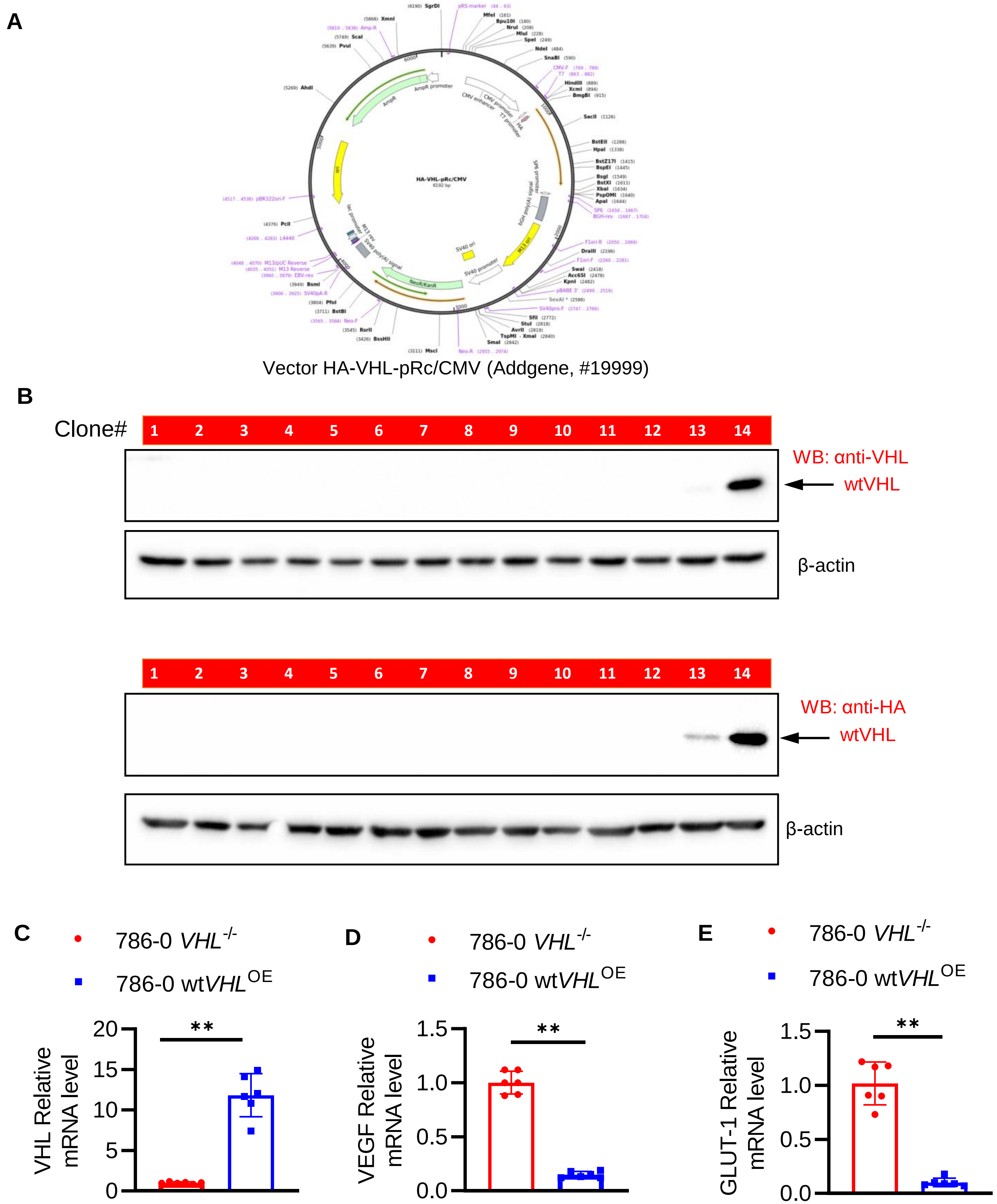

Figure S1 A Map image for vector HA-VHL-pRc/CMV. **B** VHL-overexpressed monoclonal cells were identified by western blot. **C-E** RT-qPCR analysis of VHL and downstream target genes expression (HIF-2α, VEGF, GLUT1) in VHL-isogenic RCC cell pair. Data are shown as the mean ± SD, n=3. **P < 0.01 between two indicated groups, one-way ANOVA

Figure S2

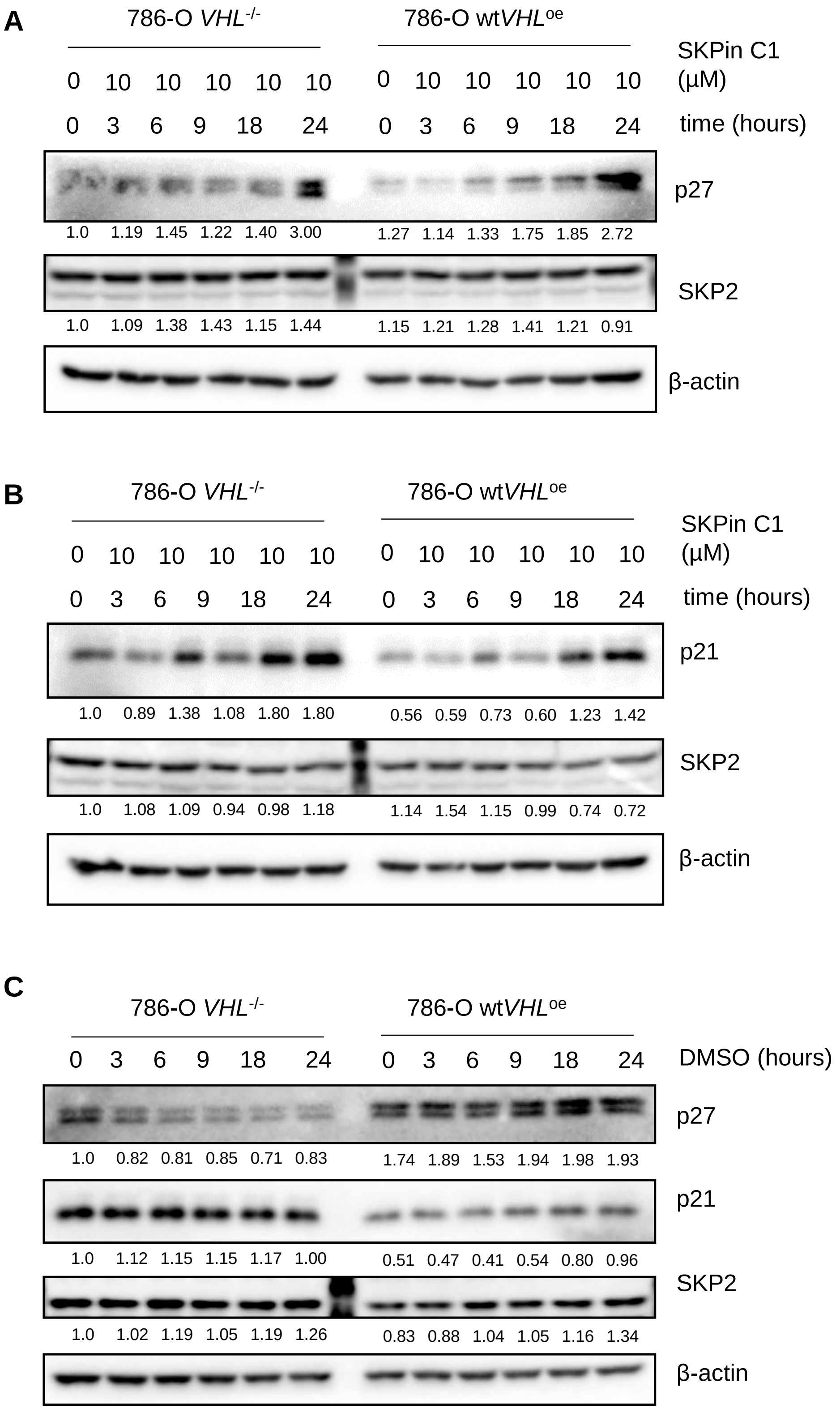

Figure S2 A-B VHL-isogenic cell pair were treated with 10 µM SKPin C1 for different times, and the protein expression of p27, p21 and SKP2 were detected by Western blotting. β-actin was used as a loading control. **C** VHL-isogenic cell pair were treated with DMSO control for different times, and the protein expression of p27, p21 and SKP2 were detected by Western blotting. β-actin was used as a loading control.

Figure S3

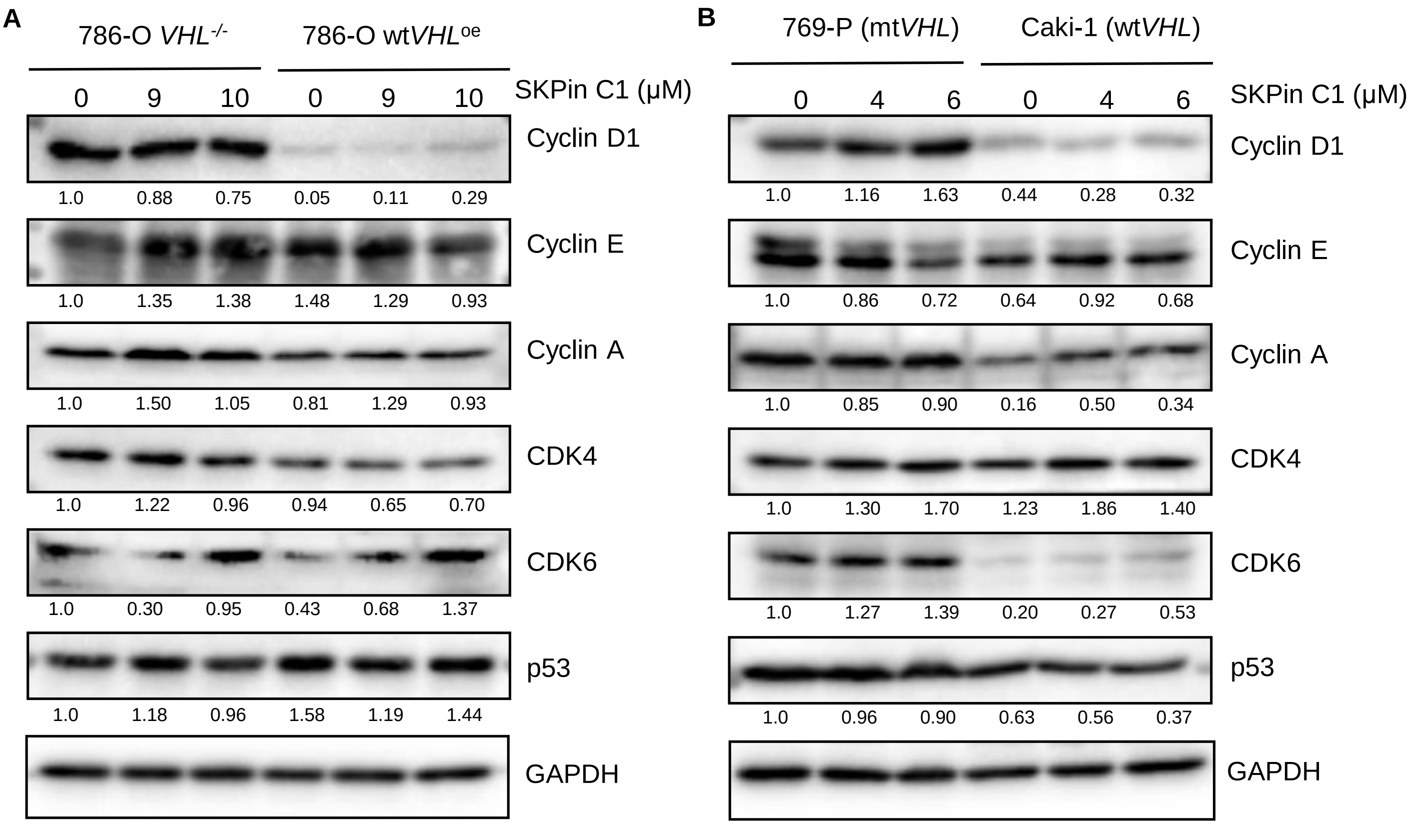

Figure S3 A-B VHL-isogenic RCC cell pair (A) and VHL non-isogenic RCC cell pair (B) were treated with indicated concentration of SKPin C1 for 24 hours. Western blot analysis of cell cycle related-proteins. GAPDH was used as a loading control.

Figure S4

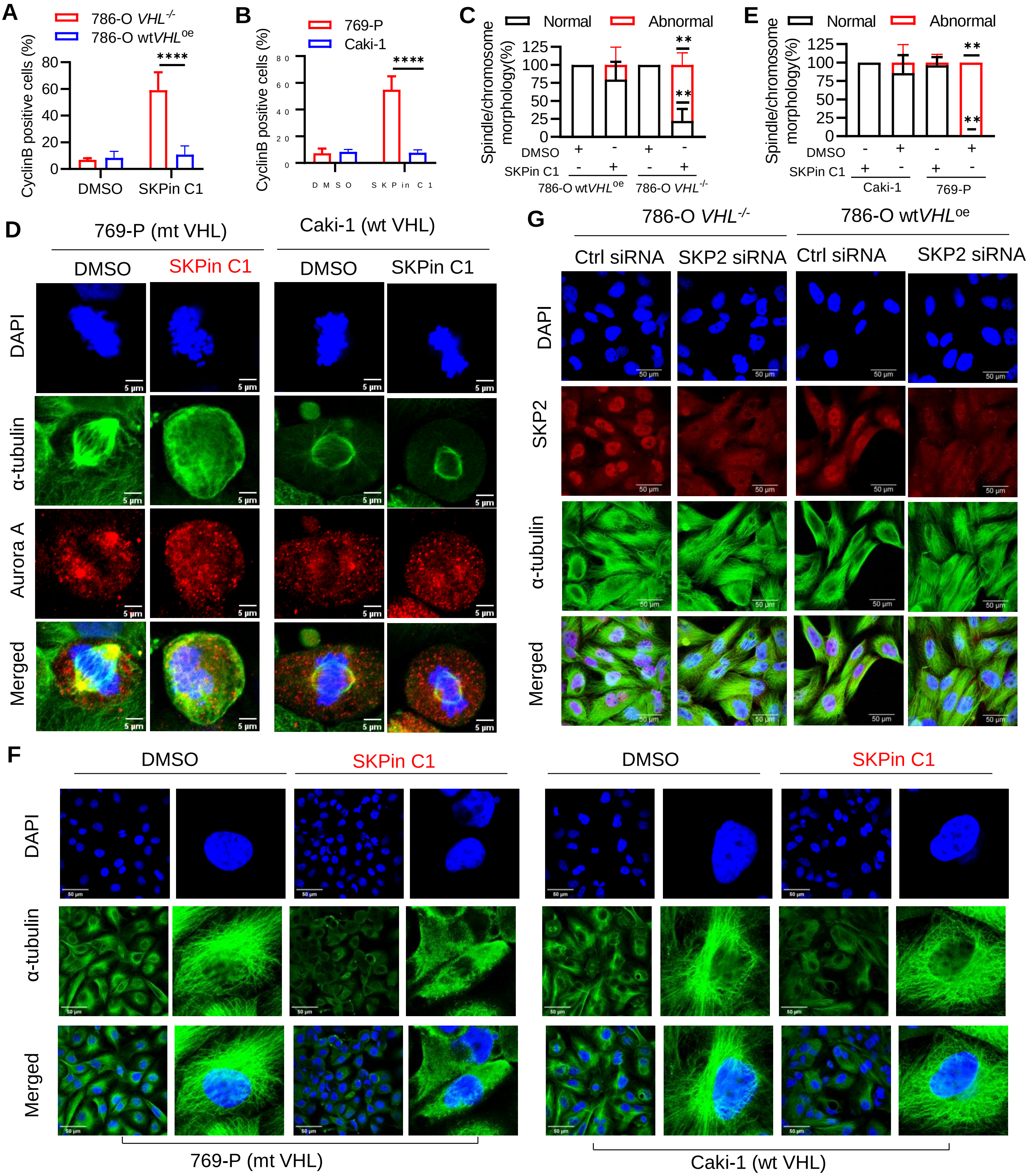

Figure S4 A-B Quantification of cyclin B positive cells in SKPin C1-treated 786-O VHL isogenic cell pair in Figure 3A-B and in VHL non-isogenic RCC cell pair in Figure 3D-E, data are shown as mean ± SD (n=3 independent experiments. For each experiment, at least 100 cells from each treatment condition were analyzed). ****P<0.0001 between two indicated groups, Student's t-test. **C** Quantification of normal spindle and abnormal spindle in SKPin C1-treated 786-O VHL isogenic cell pair, data are shown as mean ± SD (n=3 independent experiments. For each experiment, at least 100 cells from each treatment condition were analyzed). **P<0.01 between two indicated groups, Student's t-test. **D-E** VHL non-isogenic RCC cell pair were treated with 5 μM SKPin C1 for 24 hours, subsequently stained with α-tubulin antibody (green), Aurora A (red), and DAPI (blue) to show the mitotic spindle morphology (D), and the normal spindle and abnormal spindle were quantified, data are shown as mean ± SD (n=3 independent experiments. For each experiment, at least 100 cells from each treatment condition were analyzed). **P<0.01 between two indicated groups, Student's t-test (E). F VHL non-isogenic RCC cell pair were treated with 5 μM SKPin C1 for 24 hours, stained with α-tubulin antibody (green) and DAPI (blue) to show the microtubule network. Enlarged images showed the representative microtubule. **G** VHL-isogenic cell pair were transfected with 200 nM SKP2 siRNA for 48 hours, double stained with SKP2 antibody (red) and α-tubulin antibody (green) to show the effect of knockdown of SKP2 protein on microtubule morphology.

Figure S5

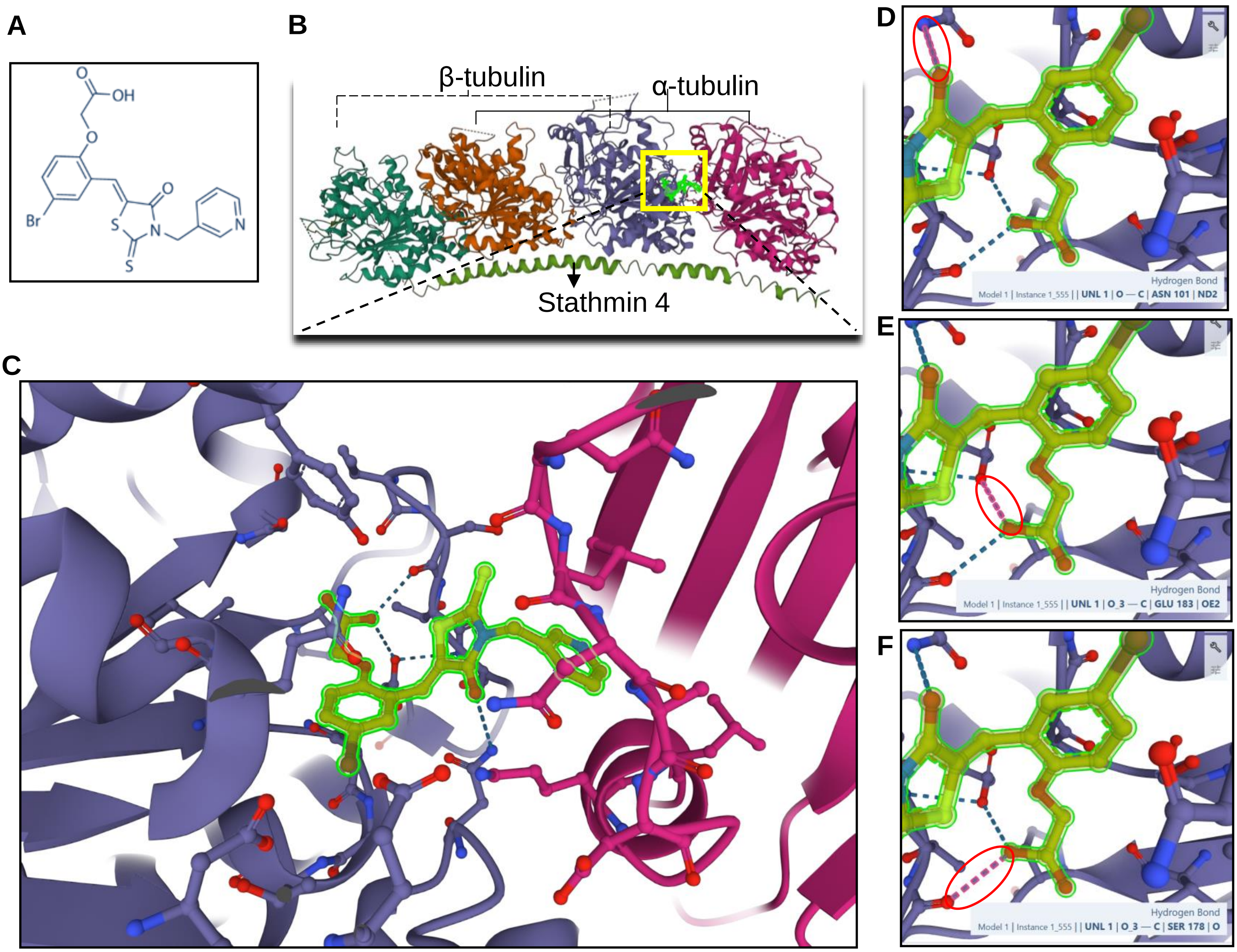

Figure S5 Docking analysis of SKPin C1 with TUBULIN-COLCHICINE STATHMIN-LIKE DOMAIN COMPLEX. Protein structures (PDB ID: SA10) were obtained from the Protein Data Bank, SKPin C1 structures (Compound CID: 5505901) were obtained from the PubChem, molecular docking study was performed by AutodockVina 1.2.2 software. **A** Structure of SKPin C1. **B** Location of the SKPin C1 in the tubulin complex. The entire tubulin complex is shown with α-tubulin (orange and pink), β-tubulin(green and purple) and stathmin 4 (light green) subunits. SKPin C1 is shown as in the yellow box. **C** SKPin C1 molecule (masked with bright green) is bound to the tubulin at the β-/α- tubulin interface. **D-F** H-bonding interaction of SKPin C1 with the β-tubulin subunit in the docked structure, shown as red ellipse.

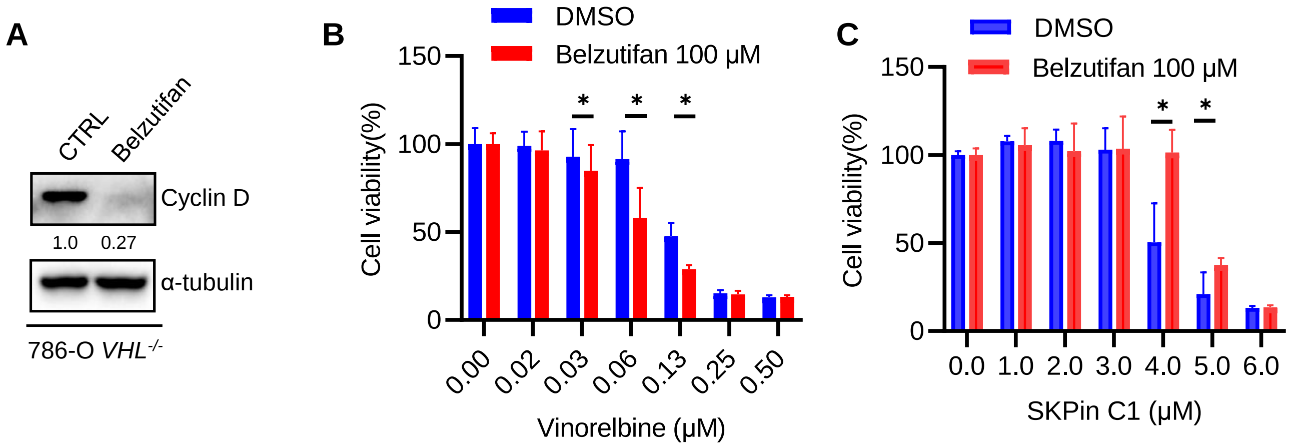

Figure S6 Effect of the cotreatment of vinorelbine or SKPin C1 with HIF-2α inhibitor belzutifan in 786-O cells. **A** Western blot analysis of the cyclin D, a downstream target gene of HIF-2α is shown. **B**-**C** The cell viability was detected by alarm blue assays. Data are shown as the mean ± SD, n=3.

Table S1

Antibodies used in this study

Antibodies	Suppliers	Cat. No	Dilution for WB	Dilution for IF
VHL	Santa Cruz Biotechnology	sc-135657	1:1000	/
HA	Cell Signaling technology	3724	1:1000	/
HIF-2α	Santa Cruz Biotechnology	sc-13596	1:500	/
VEGF	Santa Cruz Biotechnology	sc-57496	1:500	/
GLUT-1	Abcam	ab115730	1:1000	
SKP2	Cell Signaling technology	2652	1:1000	1:1000
p27	Cell Signaling technology	3686	1:1000	/
p21	Cell Signaling technology	2947S	1:1000	/
cleaved-caspase3	Cell Signaling technology	9664	1:1000	/
p-Histone H3 (Ser 10)	Santa Cruz Biotechnology	sc-8656	/	1:1000
Cyclin B1	Santa Cruz Biotechnology	sc-245	1:1000	1:1000
α-Tubulin	Abcam	ab7291	1:10000	1:1000
Acetylated Tubulin	MilliporeSigma	T7451-100UL	/	1:1000
Aurora kinase A	Cell Signaling technology	14475	1:1000	1:2000
Aurora kinase B	Abcam	ab287960	1:1000	/
Phospho-cdc25C (Ser216)	Cell Signaling technology	4901	1:1000	/
Cdc25C Antibody (H-6)	Santa Cruz Biotechnology	sc-13138	1:500	/
CDC2 P34(17)	Santa Cruz Biotechnology	sc-54	1:500	/
Phospho-cdc2 (Thr161)	Cell Signaling technology	9114	1:1000	/
GFP-Tag	Immunoway	YM3124	1:1000	/
Cyclin A	Santa Cruz Biotechnology	sc-271682	1:500	/
Cyclin D1	Santa Cruz Biotechnology	sc-20044	1:500	/
Cyclin E	Santa Cruz Biotechnology	sc-247	1:500	/
CDK4	Cell Signaling technology	12790S	1:1000	/
CDK6	Cell Signaling technology	13331S	1:1000	/
p53	Santa Cruz Biotechnology	sc-126	1:500	/
GAPDH	Santa Cruz Biotechnology	sc-47724	1:1000	/
β-actin	Santa Cruz Biotechnology,	sc-47778	1:1000	/
Anti-Tubulin-GTP (MB11)	AdipoGen	AG-27B-0009	/	1:2000
Goat anti-Mouse IgG (H+L) Secondary Antibody, HRP	Thermo Scientific	31430	1:5000	/

Table S1

Antibodies used in this study

Antibodies	Suppliers	Cat. No	Dilution for WB	Dilution for IF
Goat anti- Rabbit IgG (H+L) Secondary Antibody, HRP	Thermo Scientific	31460	1:5000	
Alexa Fluor 488 donkey anti-Mouse IgG (H+L)	Invitrogen	A21202	/	1:1000
Alexa Fluor 594 donkey anti-rabbit IgG (H+L)	Invitrogen	A21207	/	1:1000
Alexa Fluor 488 Goat anti-Human IgG (H+L)	Invitrogen	A-11013	/	1:1000

Table S2

The sequence information of the PCR primers used in this study

No.	Gene name	Suppliers	Sequences
1	GAPDH	BGI	Forward 5’-ACAACTTTGGTATCGTGGAAGG-3’
		BGI	Reverse 5’-GCCATCACGCCACAGTTTC-3'
2	VHL	BGI	Forward 5’-GGAGCCTAGTCAAGCCTGAGA-3’
		BGI	Reverse 5’-CATCCGTTGATGTGCAATGCG-3’
3	VEGF	BGI	Forward 5’-AGGGCAGAATCATCACGAAGT-3’
		BGI	Reverse 5’-AGGGTCTCGATTGGATGGCA-3’
4	GLUT-1	BGI	Forward 5’-ATTGGCTCCGGTATCGTCAAC-3’
		BGI	Reverse 5’-GCTCAGATAGGACATCCAGGGTA-3’

Table S3

Sequences of siRNAs used in this study

Gene name	Sequences
SKP2	Sense strand 5'-AUCACUUAAGUCUAGAUGGAC-3'
	Antisense strand 5'-CCAUCUAGACUUAAGUGAUAG-3'
